# Protein kinase C fusion proteins are paradoxically loss of function in cancer

**DOI:** 10.1016/j.jbc.2021.100445

**Published:** 2021-02-20

**Authors:** An-Angela N. Van, Maya T. Kunkel, Timothy R. Baffi, Gema Lordén, Corina E. Antal, Sourav Banerjee, Alexandra C. Newton

**Affiliations:** 1 Department of Pharmacology, University of California at San Diego, La Jolla, California, USA; 2 Biomedical Sciences Graduate Program, University of California at San Diego, La Jolla, California, USA

**Keywords:** protein kinase C (PKC), gene fusions, cancer, catalytic domain, loss of function, autoinhibition, constitutively active, protein degradation, regulatory domain, dominant negative, BFH, benign fibrous histiocytoma, BRCA, breast invasive carcinoma, CDH8, Cadherin-8, COSMIC, Catalogue of Somatic Mutations in Cancer, CKAR, C kinase activity reporter, DAG, diacylglycerol, LGG, low-grade glioma, LUAD, lung adenocarcinoma, LUSC, lung squamous cell carcinoma, PGNT, papillary glioneuronal tumor, PKC, protein kinase C, TCGA, The Cancer Genome Atlas, UCEC, uterine corpus endometrial carcinoma

## Abstract

Within the AGC kinase superfamily, gene fusions resulting from chromosomal rearrangements have been most frequently described for protein kinase C (PKC), with gene fragments encoding either the C-terminal catalytic domain or the N-terminal regulatory moiety fused to other genes. Kinase fusions that eliminate regulatory domains are typically gain of function and often oncogenic. However, several quality control pathways prevent accumulation of aberrant PKC, suggesting that PKC fusions may paradoxically be loss of function. To explore this topic, we used biochemical, cellular, and genome editing approaches to investigate the function of fusions that retain the portion of the gene encoding either the catalytic domain or regulatory domain of PKC. Overexpression studies revealed that PKC catalytic domain fusions were constitutively active but vulnerable to degradation. Genome editing of endogenous genes to generate a cancer-associated PKC fusion resulted in cells with detectable levels of fusion transcript but no detectable protein. Hence, PKC catalytic domain fusions are paradoxically loss of function as a result of their instability, preventing appreciable accumulation of protein in cells. Overexpression of a PKC regulatory domain fusion suppressed both basal and agonist-induced endogenous PKC activity, acting in a dominant-negative manner by competing for diacylglycerol. For both catalytic and regulatory domain fusions, the PKC component of the fusion proteins mediated the effects of the full-length fusions on the parameters examined, suggesting that the partner protein is dispensable in these contexts. Taken together, our findings reveal that PKC gene fusions are distinct from oncogenic fusions and present a mechanism by which loss of PKC function occurs in cancer.

Protein kinase C (PKC) plays a critical role in cellular homeostasis, transducing signals initiated by receptor-mediated hydrolysis of phospholipids to regulate cellular processes such as apoptosis, migration, and proliferation (1, 2, 3). With its activity finely tuned, dysregulation of PKC has been implicated in a multitude of diseases, including metabolic disorders, neurodegeneration, and most notably, cancer (4, 5, 6). Since PKC was identified as a high-affinity receptor for the tumor-promoting compounds phorbol esters in the 1980s, it was ascribed an oncogenic role (7, 8, 9). However, recent analysis of cancer-associated point mutations in PKC revealed that they are generally loss of function (10). Additionally, germline mutations in one isozyme, PKCδ, are responsible for a lymphoproliferative disorder (11, 12, 13). Furthermore, high protein levels of PKC are associated with improved survival in diverse cancers, including colon cancer (14), pancreatic cancer (15), and non-small-cell lung carcinoma (16), reframing PKC as providing a tumor suppressive function (17). Gene fusions involving PKC have also been identified in cancer. Analysis of RNA sequencing data from The Cancer Genome Atlas (TCGA) reveals that approximately 15% of in-frame kinase fusions belong to members of the AGC superfamily of serine/threonine kinases, second only to the tyrosine kinase family (18). Within the AGC kinases, the greatest abundance of gene fusions occurs in the *PRKC* genes (18).

The PKC family, encoded by the *PRKC* genes, consists of nine isozymes, categorized into three classes: conventional (α, β, γ), novel (δ, ε, η, θ), and atypical (ζ, ι/λ) (19). They all possess a C-terminal kinase domain that is constrained by a pseudosubstrate segment in the N-terminal regulatory moiety, serving to autoinhibit the enzyme in the absence of its second messengers. Release of autoinhibition occurs following engagement of the appropriate second messengers to membrane-targeting modules in the regulatory moiety. Conventional and novel isozymes of PKC are activated by binding of diacylglycerol (DAG) to one of their tandem C1 domains, with conventional isozymes also requiring binding of Ca^2+^ to their C2 domain for activation. Atypical isozymes respond to neither DAG nor Ca^2+^ but contain a protein-binding PB1 domain that mediates protein–protein interactions (20). Before PKC is catalytically competent, it is matured by a series of ordered phosphorylations that are necessary for the enzyme to adopt an autoinhibited conformation; these are the activation loop (Thr500 in PKCβII), the turn motif (Thr641 in PKCβII), and the hydrophobic motif (Ser660 in PKCβII) (21). Of these, phosphorylation of the hydrophobic motif is indispensable for autoinhibition, and dephosphorylation of this site by the PH domain leucine-rich repeat protein phosphatase PHLPP shunts PKC to degradative pathways (22). Thus, this autoinhibited conformation is dependent upon phosphorylation and is critical to ensuring PKC stability. Aberrant PKC that is not properly autoinhibited is degraded, subject to PHLPP-mediated quality control of PKC. This quality control presents a major mechanism for loss of PKC function in cancer (15). Thus, cancer-associated mutations that disrupt autoinhibitory constraints are paradoxically loss of function because the constitutively active kinase is sensitive to degradation. This raises the question as to whether cancer-associated fusions in which the pseudosubstrate is replaced by a fusion partner will be unstable or whether they have mechanisms to evade quality control degradation.

Here, we investigated PKC gene fusions that yield proteins retaining the catalytic domain of PKC (3' PKC fusions) as well as fusions that yield proteins retaining the regulatory domain of PKC (5' PKC fusions). Cell-based assays revealed that the overexpressed catalytic domain fusions were constitutively active but unable to retain processing phosphorylations. Thus, these fusions were unstable. Utilizing CRISPR/Cas9-mediated gene editing to encode an endogenous 3' PKC fusion, we were able to detect the fusion at the mRNA level in CRISPR-edited cells but not at the protein level, consistent with the fusion protein being too unstable to accumulate in cells. Analysis of a 5' PKC fusion encoding the regulatory domain of PKC, with complete loss of the kinase domain, revealed that it functioned in a dominant-negative manner to suppress endogenous PKC activity. Taken together, these results identify PKC fusions as a mechanism for loss of PKC function in cancer.

## Results

### A multitude of PKC fusions have been identified in cancer

**Figure 1:**
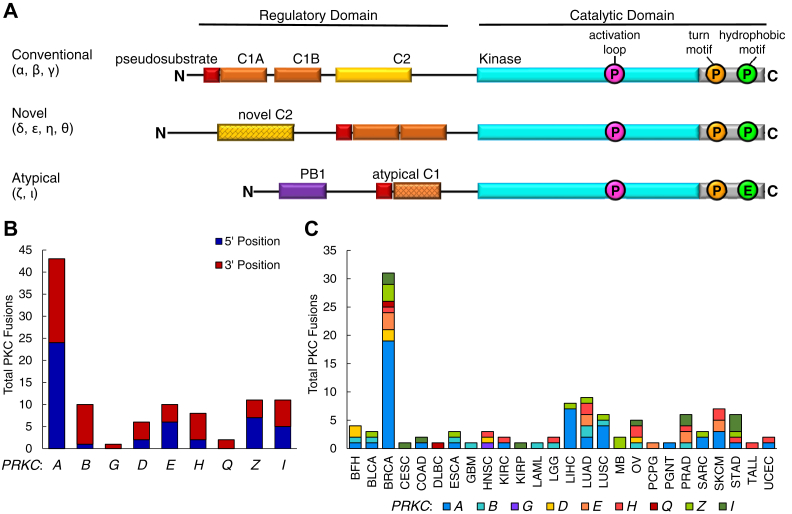
**Fusions of the protein kinase C regulatory and catalytic moieties are detected in a multitude of cancers.***A*, domain structure of the three PKC subclasses, including pseudosubstrate (*red*), C1 domains (*orange*), C2 domain (*yellow*), and kinase domain (*cyan*). PKC structure consists of a variable N-terminal regulatory domain followed by a conserved C-terminal catalytic domain. The activation loop, turn motif, and hydrophobic motif phosphorylation sites necessary for catalytic competency are indicated (*pink*, *orange*, and *green*, respectively). *B*, curated from the literature, the Mitelman Database of Chromosome Aberrations and Gene Fusions in Cancer, the TCGA Tumor Fusion Gene Data Portal, ChimerDB v3.0, and COSMIC, number of 5' and 3' gene fusions for each PKC isozyme is depicted. *C*, curated PKC fusions in (*B*) categorized by cancer type in which they were detected. BFH, benign fibrous histiocytoma; BLCA, bladder urothelial carcinoma; BRCA, breast invasive carcinoma; CESC, cervical squamous cell carcinoma and endocervical adenocarcinoma; COAD, colon adenocarcinoma; DLBC, lymphoid neoplasm diffuse large B-cell lymphoma; ESCA, esophageal carcinoma; GBM, glioblastoma multiforme; HNSC, head and neck squamous cell carcinoma; KIRC, clear cell renal cell carcinoma; KIRP, kidney renal papillary cell carcinoma; LAML, acute myeloid leukemia; LGG, low-grade glioma; LIHC, liver hepatocellular carcinoma; LUAD, lung adenocarcinoma; LUSC, lung squamous cell carcinoma; MB, medulloblastoma; OV, ovarian serous cystadenocarcinoma; PCPG, pheochromocytoma and paraganglioma; PGNT, papillary glioneuronal tumor; PRAD, prostate adenocarcinoma; SARC, sarcoma; SKCM, skin cutaneous melanoma; STAD, stomach adenocarcinoma; TALL, T-cell acute lymphoblastic leukemia; UCEC, uterine corpus endometrial carcinoma.

In order to assess the landscape of PKC fusions in cancer, we queried the literature and several online databases to curate a comprehensive list of all PKC gene fusions identified from patient tumor samples (Table S1) (18, 23, 24, 25, 26, 27, 28, 29, 30, 31, 32, 33, 34, 35, 36, 37). The online databases utilized in this study include the Mitelman Database of Chromosome Aberrations and Gene Fusions in Cancer, the TCGA Tumor Fusion Gene Data Portal, ChimerDB v3.0, and the Catalogue of Somatic Mutations in Cancer (COSMIC) (30, 38, 39, 40). With this list of fusions, we were able to ascertain that each of the nine isozymes from the three subclasses of the PKC family (Fig. 1*A*) was detected in at least one gene fusion (Fig. 1*B*). Although there were only seven isozymes for which there were fusions detected that retained the 5' end of PKC, there were fusions detected for all isozymes where the 3' end of PKC was retained. The greatest number of fusions was detected for *PRKCA* with 43 fusions identified, and the majority of these (19 fusions) were found in breast cancer samples. In total, 102 fusions were identified across all PKC family members. PKC gene fusions were detected in 26 different cancer types, from benign fibrous histiocytoma (BFH) to uterine corpus endometrial carcinoma (UCEC) (Fig. 1*C*). Among these different cancer types, the greatest number of PKC gene fusions was identified in breast invasive carcinoma (BRCA) with 31 fusions detected and lung adenocarcinoma (LUAD) with nine fusions detected. Notably, approximately half of these fusions resulted in products that were out of frame (Table S1). Several of the fusions were detected in different cancer types, and a few fusions were recurrent (Table S2). Given that PKC is the most abundantly fused AGC kinase and the fusions were detected in a multitude of cancers, we sought to determine how these fusions might affect PKC signaling and contribute to tumorigenesis. To this end, we selected three 3' fusions (*TANC2-PRKCA*, *SLC44A1-PRKCA*, and *GGA2-PRKCB*) and one 5' fusion (*PRKCA-CDH8*) in this study for further analysis (18, 34, 35, 36, 37, 41).

### PKC catalytic domain fusions are unphosphorylated yet constitutively active

**Figure 2:**
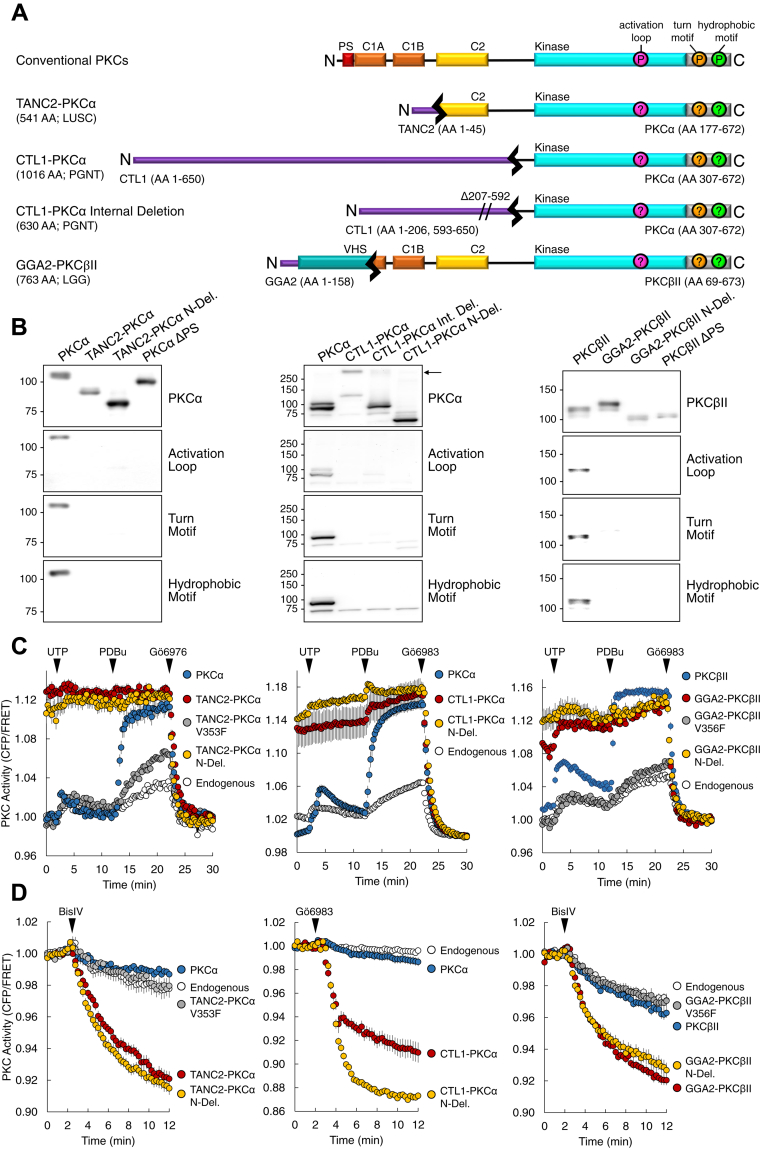
**3' PKC fusions yield proteins that are unphosphorylated at the PKC priming sites yet constitutively active.***A*, schematic of wild-type and PKC fusion constructs used in this study. *B*, immunoblot analysis of lysates from COS7 cells transfected with the indicated YFP-tagged constructs. Blots probed with the indicated total PKC or phospho-specific antibodies. *Arrow* indicates border between stacking and separating gel. Data are representative of three independent experiments. *C*, analysis of agonist-induced PKC activity in COS7 cells transfected with CKAR alone (Endogenous) or cotransfected with the indicated mCherry-tagged PKC construct. Cells treated with agonists UTP (100 μM), PDBu (200 nM), and either inhibitor Gӧ6976 or Gӧ6983 (1 μM). Graphs depict normalized FRET ratio changes (mean ± SEM) from three independent experiments. *D*, analysis of basal PKC activity in COS7 cells transfected with CKAR alone (Endogenous) or cotransfected with the indicated mCherry-tagged PKC construct. Cells treated with inhibitor BisIV (2 μM) or Gӧ6983 (1 μM). Graphs depict normalized FRET ratio changes (mean ± SEM) from three independent experiments.

3' PKC gene fusions yield proteins in which the N-terminal regulatory domain of PKC is truncated and replaced with the N-terminal domain of its gene partner. The 3' fusions *TANC2-PRKCA*, *SLC44A1-PRKCA*, and *GGA2-PRKCB*, found in lung squamous cell carcinoma (LUSC), papillary glioneuronal tumors (PGNT), and low-grade glioma (LGG), respectively, were selected for biochemical analysis (Fig. 2*A*) (34, 35, 36, 37). *TANC2-PRKCA* encodes the first 45 amino acid residues of TANC2 fused to the last 496 residues of PKCα, and *SLC44A1-PRKCA* encodes the first 650 residues of CTL1 fused to the last 366 residues of PKCα. *GGA2-PRKCB* encodes the first 158 residues of GGA2 fused to the last 605 residues of PKCβII. TANC2 has been identified as a scaffolding protein in dendritic spines, and CTL1 functions as a choline transporter (42, 43, 44). GGA2 is part of the Golgi-localized, gamma adaptin ear-containing, ARF-binding (GGA) family of adaptor proteins, which localize to the trans-Golgi network and regulate protein trafficking (45). The protein products resulting from the fusion transcripts retain the complete catalytic domain of PKC with some portion of the regulatory domain. Specifically, TANC2-PKCα retains the C2 domain and GGA2-PKCβII retains the C1B and C2 domains, whereas CTL1-PKCα only retains part of the hinge between the regulatory and catalytic moieties. Notably, the autoinhibitory pseudosubstrate, located at the N terminus, is lost in all three fusion proteins. In our biochemical analysis of these catalytic domain fusions, we also analyzed N-deletion proteins in which we delete the fusion partner and retain only the PKC portion of the fusion. Additionally, we analyzed a variant of the CTL1-PKCα fusion protein in which there was an internal deletion within CTL1 (35).

Phosphorylation at the three PKC priming sites, the activation loop, turn motif, and hydrophobic motif, is necessary for catalytic competency. Thus, we determined whether the fusion proteins and their variants are also phosphorylated at these sites. Whereas wild-type PKC was constitutively phosphorylated at all three processing sites, the full-length fusion proteins TANC2-PKCα, CTL1-PKCα, and GGA2-PKCβII were not (Fig. 2*B*). The PKCα and PKCβII N-deletion proteins, comprising only the PKC portion of the fusion, as well as the CTL1-PKCα internal deletion (Int. Del.) protein, were also not appreciably phosphorylated at the activation loop, turn motif, or hydrophobic motif; neither were constructs in which the pseudosubstrate was deleted (ΔPS). We have previously reported that mutations that impair autoinhibition of PKC prevent retention of phosphate at the priming sites (15). Lack of phosphorylation at the priming sites of these proteins is consistent with that of PKCα and PKCβII proteins where the pseudosubstrate is deleted (ΔPS). In summary, both the full-length fusion proteins and the N-deletion proteins lacked phosphorylation at the priming sites.

We next addressed whether the fusion proteins exhibited catalytic activity, as assessed with the genetically encoded, fluorescence resonance energy transfer (FRET)-based reporter for PKC activity, C kinase activity reporter (CKAR) (46). Stimulation of COS7 cells expressing mCherry-tagged wild-type PKC (blue) resulted in a transient increase in CKAR phosphorylation upon treatment with the physiological agonist UTP, which generates Ca^2+^ and DAG. Subsequent treatment with the phorbol ester PDBu resulted in maximal, sustained activity, which was abolished upon addition of PKC inhibitor, either Gӧ6976 or Gӧ6983 (Fig. 2*C*). In contrast, the full-length fusion proteins (red) were maximally active under basal conditions and did not respond to the addition of UTP or PDBu. However, addition of inhibitor reduced activity to baseline levels observed following inhibition of wild-type enzyme. Introduction of a mutation to render the PKC kinase domain catalytically dead (V353F in PKCα and V356F in PKCβ, gray) abolished the constitutive activity of the full-length fusion proteins (47, 48). Moreover, similar results were obtained for the N-deletion proteins that comprised only the PKC segment of the fusions (N-Del., yellow). To further probe the basal activity of the fusion proteins, COS7 cells expressing either CKAR alone (white) or coexpressing mCherry-tagged PKC were treated with a PKC inhibitor alone (BisIV or Gӧ6983) (Fig. 2*D*). Inhibitor treatment resulted in only a modest reduction in PKC activity for cells expressing wild-type PKC (blue), consistent with effective autoinhibition in the absence of second messengers. In contrast, addition of inhibitor in cells overexpressing the full-length fusion proteins (red) or the PKC component of the fusions (yellow) resulted in a robust reduction in CKAR phosphorylation, indicating high basal activity. This activity was abolished when introducing the mutation rendering the kinase domain catalytically dead (gray). Thus, although the full-length fusion and N-deletion proteins were unphosphorylated at the PKC priming sites, they exhibited constitutive, agonist-independent activity.

Phosphorylation at the hydrophobic motif is necessary for PKC maturation, leading us to ask whether the fusion proteins had been phosphorylated during their maturation but were unable to retain the phosphate because of their inability to autoinhibit. Mutation of the hydrophobic motif Ser to Ala in TANC2-PKCα (S657A in wild-type PKCα, pink) or GGA2-PKCβII (S660A in wild-type PKCβII, pink) reduced basal activity to levels comparable with those of wild-type PKC (Fig. S1, *A* and *B*, respectively). In contrast, mutation of the turn motif in either fusion protein to the phosphomimetic residue Glu (T638E in wild-type PKCα and T641E in wild-type PKCβII, green) resulted in expression of proteins with high basal activity. Mutation of the hydrophobic motif in TANC2-PKCα to Glu (S657E in wild-type PKCα, orange) also resulted in protein with high basal activity. The lack of significant activity with fusions containing an Ala at the hydrophobic motif is consistent with previous studies indicating that phosphorylation at this site during PKC maturation is necessary for activity (15). To confirm that these fusions have the intrinsic ability to become phosphorylated at this site, the GGA2-PKCβII fusion was expressed in insect cells, a system where low PHLPP activity permits retention of phosphate on aberrant PKC (49). Like wild-type PKCβII, GGA2-PKCβII fusion protein purified from baculovirus-infected insect cells was phosphorylated at all three processing sites (Fig. S1*C*). This is consistent with lack of phosphate at these sites in mammalian cells resulting from the previously described quality control mechanism mediated by PHLPP.

### Fusions of the PKC catalytic domain are unstable

**Figure 3:**
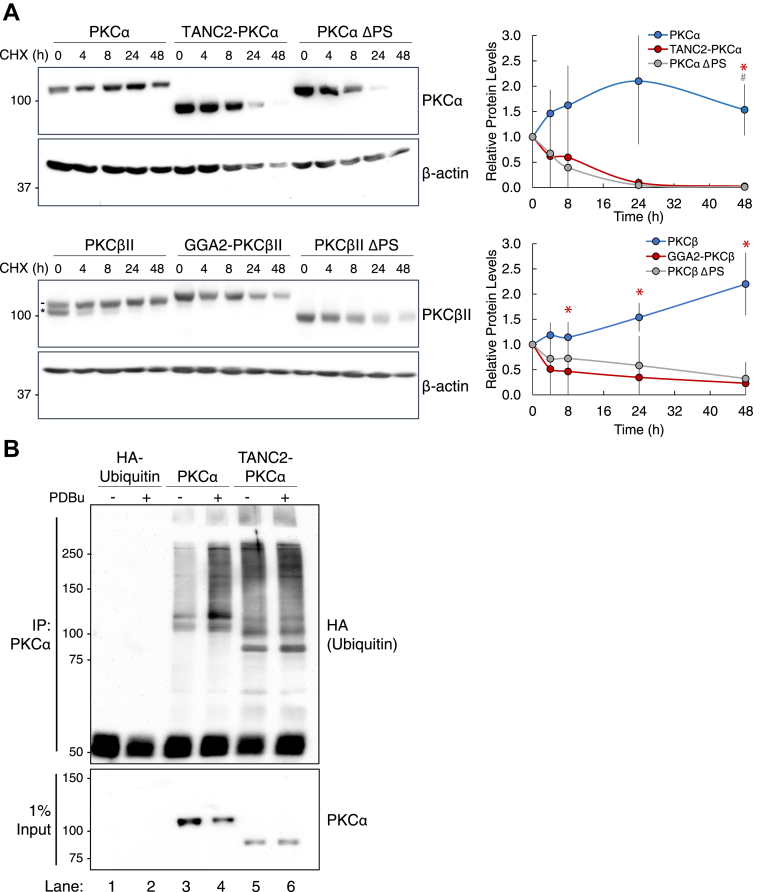
**The open conformation that renders PKC catalytic domain fusions constitutively active also causes them to be markedly unstable.***A*, immunoblot analysis of lysates from COS7 cells transfected with the indicated YFP-tagged constructs, treated with cycloheximide (CHX, 355 μM), and harvested at the indicated timepoints. Blots probed for indicated PKC and β-actin. For wild-type PKC, *dash* depicts slower mobility phosphorylated PKC while *asterisk* depicts faster mobility unphosphorylated PKC. For quantification, PKC protein levels normalized to loading control and then to t = 0. Blots are representative, and graphs depict mean ± SEM from three independent experiments. ∗*p* < 0.05 (Student’s *t-*test). *Asterisk* indicates significance between wild-type PKC and the fusion, and # indicates significance between wild-type PKC and PKC ΔPS. *B*, immunoblot analysis of PKCα immunoprecipitates from COS7 cells transfected with HA-tagged ubiquitin alone or cotransfected with indicated YFP-tagged PKC and treated with PDBu (200 nM) or vehicle (DMSO) for 30 min. Blots probed for HA to assess ubiquitination of wild-type PKC compared with the fusion. Data are representative of three independent experiments.

Prolonged activation of PKC, such as that which occurs with phorbol ester treatment, ultimately results in PKC downregulation and degradation (50, 51). To determine whether the constitutively active PKC fusion proteins are also sensitive to degradation, COS7 cells overexpressing YFP-tagged wild-type PKC, fusion protein, or pseudosubstrate-deleted PKC were treated with cycloheximide to inhibit protein synthesis, and degradation of the existing PKC protein was monitored (Fig. 3*A*). Wild-type PKCα and PKCβII degraded slowly, with over half the protein remaining after 48 h treatment with cycloheximide. In contrast, the TANC2-PKCα and GGA2-PKCβII fusion proteins turned over more rapidly, with little to no protein detected at 24 h. Deletion of the pseudosubstrate (ΔPS) in both PKCα and PKCβII resulted in rapid turnover, comparable with that observed for the fusion proteins. Note that the minor species of unphosphorylated wild-type PKCβII (Fig. 3*A*, lower panel, asterisk) was rapidly degraded, similar to that of the fusion and ΔPS proteins. Immunoblot analysis of GGA2-PKCβII fusion protein purified from HEK 293T cells revealed that it was associated with the molecular chaperones Hsp90, Hsp70, and Cdc37 (Fig. S2, *A* and *B*). This suggests that the fusion protein maintains an open conformation that permits binding of these chaperones (52, 53, 54). Thus, these data demonstrate that the fusion proteins are intrinsically unstable, consistent with our previous studies revealing that lack of phosphate at the hydrophobic motif targets PKC for degradation.

Given that the fusion proteins were observed to be more sensitive to degradation, we next asked whether they were more ubiquitinated than wild-type PKC. To address this, we performed a cellular ubiquitination assay in which we overexpressed ubiquitin alone or in conjunction with wild-type PKCα or the TANC2-PKCα fusion in COS7 cells, immunoprecipitated the PKC protein, and assessed the degree of ubiquitination (Fig. 3*B*). Additionally, we compared cells treated with the phorbol ester PDBu to vehicle (DMSO), as treatment with phorbol esters promotes ubiquitination and ultimately results in PKC degradation. In basal conditions, TANC2-PKCα was significantly more ubiquitinated than wild-type PKCα (lane 5 versus lane 3). Treatment with PDBu induced ubiquitination of wild-type PKCα but had no effect on the ubiquitination of the fusion protein, which lacks the domains necessary to bind PDBu. These results indicate that the unstable fusion protein is more ubiquitinated compared with wild-type PKC, in accordance with its enhanced sensitivity to degradation.

### CRISPR-edited cells exhibit detectable PKC fusion mRNA but not protein

**Figure 4:**
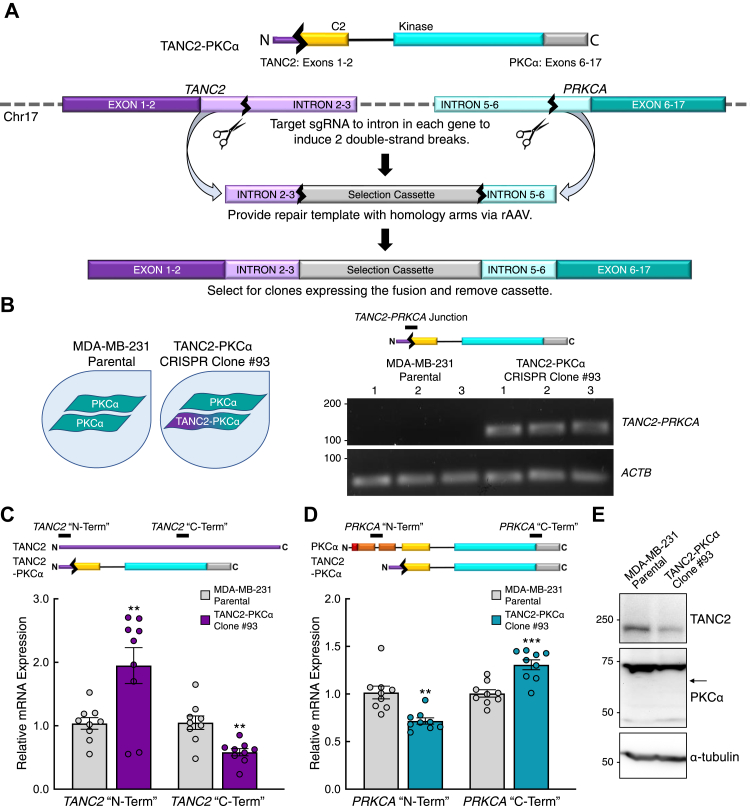
**The *TANC2-PRKCA* fusion is detected at the mRNA level but not the protein level in CRISPR-edited cells.***A*, schematic illustrating strategy to generate cells expressing the *TANC2-PRKCA* fusion *via* CRISPR/Cas9 gene editing. *B*, mRNA expression of the *TANC2-PRKCA* fusion and actin (*ACTB*) in the parental and CRISPR-edited clone analyzed by RT-PCR. Product indicated by black bar on schematic. Reactions analyzed on agarose gel, with each lane representing an individual replicate. *C*, *TANC2* mRNA expression at the 5' (“N-Term”) and 3' (“C-Term”) ends in the parental and CRISPR-edited clone analyzed by RT-qPCR and normalized to actin (*ACTB*). Products indicated by black bars on schematic. Data represent means ± SEM from at least three independent experiments. ∗∗*p* < 0.01 (Student’s *t-*test). *D*, *PRKCA* mRNA expression at the 5' (“N-Term”) and 3' (“C-Term”) ends in the parental and CRISPR-edited clone analyzed by RT-qPCR and normalized to actin (*ACTB*). Products indicated by black bars on schematic. Data represent means ± SEM from at least three independent experiments. ∗∗*p* < 0.01, ∗∗∗*p* < 0.001 (Student’s *t-*test). *E*, immunoblot analysis of lysates from parental and CRISPR-edited cells, probed for TANC2, PKCα, and α-tubulin. *Arrow* indicates predicted mobility of the fusion protein. Data are representative of three independent experiments.

Given the instability of the catalytic domain fusion proteins, we sought to determine whether they would be detected when expressed at the endogenous locus. To accomplish this, we utilized CRISPR/Cas9-mediated gene editing to engineer the *TANC2-PRKCA* fusion from the endogenous genes in the MDA-MB-231 breast cancer cell line (Fig. 4*A*) (55, 56). Employing two guide RNAs, one directed at an intron of *TANC2* and one directed at an intron of *PRKCA*, we generated a heterozygous clone (CRISPR Clone #93) with one wild-type allele of *PRKCA* and one allele of the *TANC2-PRKCA* fusion. We first validated expression of *TANC2-PRKCA* at the mRNA level by amplifying a product at the fusion junction. The fusion mRNA was detected in cells of the CRISPR-edited clone but not the parental cell line (Fig. 4*B*). Next, we examined expression of *TANC2* and *PRKCA*, targeting the 5' and 3' ends of each mRNA, which would correspond to the respective “N-Term” and “C-Term” of the resulting proteins. Compared with the parental MDA-MB-231 cells, the CRISPR-edited cells exhibited an increase in expression of the *TANC2* “N-Term” product and a decrease in expression of the “C-Term” product (Fig. 4*C*). A decrease in expression of the *TANC2* “C-Term” product is in concordance with the fusion-expressing cells containing only one copy of wild-type *TANC2*, whereas the increase in expression of the *TANC2* “N-Term” product may reflect compensatory upregulation from loss of the other copy of *TANC2*. Probing for *PRKCA* expression in the parental and CRISPR-edited cells revealed the reverse; expression of the *PRKCA* “N-Term” product was decreased in the fusion-expressing cells, whereas expression of the “C-Term” product was increased (Fig. 4*D*). In this case, the decrease in expression of the “N-Term” product in the fusion-expressing cells can be attributed to loss of a copy of wild-type *PRKCA*, and as with the *TANC2* “N-Term” product, the increase in expression of the *PRKCA* “C-Term” product may reflect compensatory upregulation. Consistent with upregulation of the wild-type alleles, increased *PRKCA* expression was reported in the tumor in which the fusion was originally detected (34).

Upon confirmation of *TANC2-PRKCA* expression in the CRISPR-edited cells, we next assessed whether the fusion could be detected at the protein level. Both TANC2 and PKCα protein were readily detected in the parental MDA-MB-231 cells and in the cells of the fusion-expressing clone, with lower expression in the fusion-expressing clone consistent with loss of one allele (Fig. 4*E*). Importantly, the TANC2-PKCα fusion protein, predicted to migrate at 65 kDa, was not detected in the CRISPR-edited cells (expected position indicated by arrow). This suggests that the fusion protein is indeed too unstable to appreciably accumulate in an endogenous context. We then sought to determine whether degradation of the fusion protein could be slowed. Inhibition of proteasomal (Fig. S3*A*) or endosomal/lysosomal (Fig. S3*B*) degradative pathways in our overexpression studies with cycloheximide did not slow degradation of the TANC2-PKCα fusion protein. Additionally, knockdown of RINCK, the E3 ligase that catalyzes ubiquitin-mediated degradation of wild-type PKC, as well as CHIP, a “quality control” E3 ligase that cooperates with molecular chaperones to ubiquitinate unfolded proteins, did not allow for accumulation of the protein in the CRISPR-edited cells (Fig. S4) (57, 58). These data thus suggest that the unstable fusion protein is degraded through a separate, proteasome- and endosome-independent mechanism. Importantly, the fusion protein is paradoxically loss of function as it is unable to accumulate to detectable levels in cells in an endogenous context.

### CRISPR-edited fusion cells exhibit reduced apoptosis

**Figure 5:**
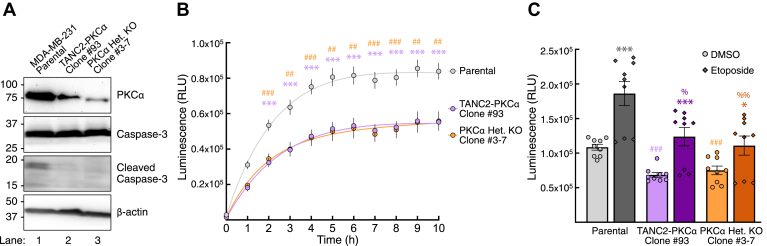
***TANC2-PRKCA*-expressing cells exhibit reduced levels of cleaved caspase-3 and decreased apoptosis.***A*, immunoblot analysis of lysates from parental, CRISPR-edited TANC2-PKCα fusion, and heterozygous PKCα knockout cells, probed for caspase-3, cleaved caspase-3, PKCα, and β-actin. Data are representative of three independent experiments. *B*, apoptosis assay performed on MDA-MB-231 parental (*gray*), CRISPR-edited TANC2-PKCα fusion (*purple*), or heterozygous PKCα knockout (*orange*) cells. Data represent luminescence upon binding of annexin V-luciferase to cell-surface-exposed phosphatidylserine as a function of time following addition of reagents. Graph depicts mean ± SEM from three independent experiments. ∗∗*p* < 0.01, ∗∗∗*p* < 0.001 (Student’s *t-*test). *Asterisk* indicates significance between parental and fusion-expressing cells, and # indicates significance between parental and heterozygous PKCα knockout cells. *C*, apoptosis assay described in (*B*) was performed on MDA-MB-231 parental (*gray*), CRISPR-edited TANC2-PKCα fusion (*purple*), or heterozygous PKCα knockout (*orange*) cells treated with etoposide (12.5 μM) or vehicle (DMSO) for 24 h. Graph depicts mean ± SEM from three independent experiments. ∗*p* < 0.05, ∗∗*p* < 0.01, ∗∗∗*p* < 0.001 (Student’s *t-*test). *Asterisk* indicates significance between vehicle and etoposide-treated conditions for each cell line. # and % indicate significance between the parental and CRISPR-edited fusion or heterozygous PKCα knockout cells in basal and etoposide-treated conditions, respectively.

To determine the functional relevance of the catalytic domain fusion, whose expression would invariably result in the loss of PKC activity, we addressed whether there was any difference in apoptosis between the parental MDA-MB-231 cells and the CRISPR-edited fusion cells. The initiation of programmed cell death is characterized by proteolytic cleavage and consequent activation of proteases such as caspase-3 (59, 60). We thus assessed basal apoptosis in the two cell lines by probing for levels of cleaved caspase-3 (Fig. 5*A*). Compared with the parental cells, cells expressing the *TANC2-PRKCA* fusion exhibited decreased levels of cleaved caspase-3 (lane 1 *versus* lane 2). To determine whether this resulted from loss of PKC, we also generated a heterozygous knockout of PKCα in the MDA-MB-231 cell line and assessed levels of cleaved caspase-3. As with the fusion-expressing cells, the heterozygous PKCα knockout cells had reduced levels of cleaved caspase-3 compared with parental cells (lane 3 versus lane 1). This suggests that the decreased cleaved caspase-3 levels observed in the fusion-expressing line resulted from loss of PKC activity. To confirm that the decreased cleaved caspase-3 reflected decreased apoptosis, we performed an apoptosis assay that measures cell-surface-exposed phosphatidylserine, detected by luminescence of bound annexin V-luciferase (Fig. 5*B*). Over the course of 10 h, both the fusion-expressing (purple) and heterozygous knockout (orange) cells exhibited significantly lower levels of apoptosis compared with the parental cells (gray), with similar levels of apoptosis between the CRISPR-edited lines. We also assessed apoptosis in the cell lines following treatment with etoposide or vehicle (DMSO) for 24 h (Fig. 5*C*). Whereas etoposide significantly increased apoptosis in all three cell lines compared with their respective vehicle-treated controls, the fusion cells and heterozygous knockout cells continued to have significantly lower levels of apoptosis compared with the parental cells. Etoposide-induced apoptosis in the fusion and heterozygous knockout cells reached levels comparable with that of the vehicle-treated parental cells (basal apoptosis). Altogether, these results indicate that expression of the 3' PKC fusion and subsequent loss of PKC activity suppress basal apoptosis, conditions that would promote a hallmark of cancer—cell survival.

### PKC regulatory domain fusions are LOF and potentially dominant-negative

**Figure 6:**
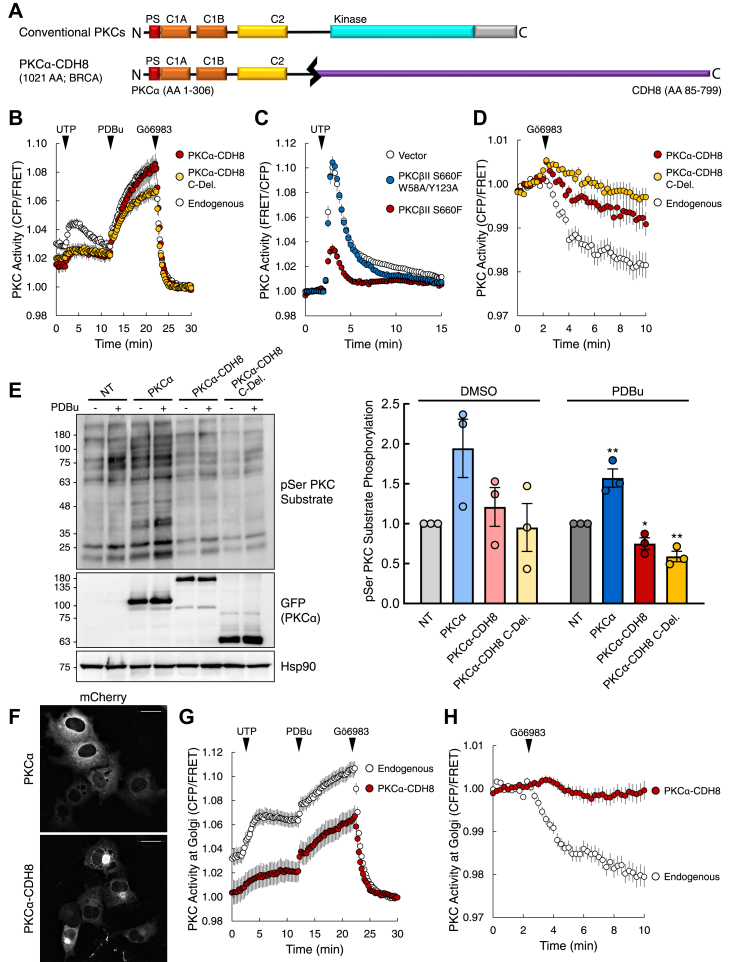
**5' PKC fusions act in a dominant-negative manner, suppressing the activity of wild-type PKC.***A*, schematic of wild-type and PKCα-CDH8 fusion construct used in this study. In addition to the full-length fusion, a C-deletion construct in which the C-terminal fusion partner is deleted was also generated (PKCα C-Del.). *B*, analysis of agonist-induced PKC activity in COS7 cells transfected with CKAR alone (Endogenous) or cotransfected with the indicated mCherry-tagged PKC construct. Cells treated with agonists UTP (100 μM), PDBu (200 nM), and inhibitor Gӧ6983 (1 μM). Graphs depict normalized FRET ratio changes (mean ± SEM) from three independent experiments. *C*, analysis of agonist-induced PKC activity in COS7 cells transfected with the reporter CKAR2 and either vector or the indicated mCherry-tagged PKC construct. Cells treated with UTP (100 μM). Graphs depict normalized FRET ratio changes (mean ± SEM) from three independent experiments. *D*, analysis of basal PKC activity in COS7 cells transfected with CKAR alone (Endogenous) or cotransfected with the indicated mCherry-tagged PKC construct. Cells treated with inhibitor Gӧ6983 (1 μM). Graphs depict normalized FRET ratio changes (mean ± SEM) from four independent experiments. *E*, immunoblot analysis of lysates from COS7 cells transfected with the indicated YFP-tagged PKC and treated with PDBu (200 nM) or vehicle (DMSO) for 30 min. Blots probed with antibodies for pSer PKC substrate and GFP. Quantification of pSer PKC substrate signal normalized to loading control (Hsp90) depicts mean ± SEM from three independent experiments. ∗*p* < 0.05, ∗∗*p* < 0.01 (Student’s *t-*test). *F*, images of cells from (*B*) prior to drug addition depicting mCherry fluorescence for wild-type PKCα (*top*) and the PKCα-CDH8 fusion (*bottom*). Scale bar, 20 μm. *G*, analysis of agonist-induced PKC activity at the Golgi in COS7 cells transfected with Golgi-targeted CKAR alone (Endogenous) or cotransfected with mCherry-tagged PKCα-CDH8. Cells treated with agonists UTP (100 μM), PDBu (200 nM), and inhibitor Gӧ6983 (1 μM). Graphs depict normalized FRET ratio changes (mean ± SEM) from three independent experiments. *H*, analysis of basal PKC activity at the Golgi in COS7 cells transfected with Golgi-targeted CKAR alone (Endogenous) or cotransfected with mCherry-tagged PKCα-CDH8. Cells treated with inhibitor Gӧ6983 (250 nM). Graphs depict normalized FRET ratio changes (mean ± SEM) from three independent experiments.

Whereas PKC catalytic domain fusions are loss of function because of their inherent sensitivity to degradation, regulatory domain fusions are intrinsically loss of function because the portion of the gene encoding the C-terminal kinase domain is truncated. However, expression of these 5' PKC fusions may have functional consequences beyond loss of the catalytic domain. Previous studies investigating loss-of-function mutations in PKC demonstrated that kinase-dead mutants may act in a dominant-negative manner (10). Thus, we addressed whether 5' PKC fusions, retaining various elements of the regulatory moiety, can also be dominant-negative. For our biochemical analyses, we selected the fusion *PRKCA-CDH8*, originally detected in breast cancer (Fig. 6*A*) (18, 30, 41). *PRKCA-CDH8* translates to a protein containing the first 306 amino acid residues of PKCα fused to the last 715 residues of Cadherin-8 (CDH8). CDH8 is a type II classical cadherin that mediates cell–cell adhesion and regulates proliferation of cortical interneurons (61). The portion of *PRKCA* retained in the fusion encodes the entire PKC regulatory moiety, including the inhibitory pseudosubstrate, the C1A and C1B domains, and the C2 domain. In addition to analyzing the full-length fusion, we also analyzed a C-deletion (C-Del.) variant containing only the PKC portion of the fusion. First, we determined the effect of the regulatory fusion and the C-deletion variant on endogenous PKC activity utilizing the genetically encoded FRET reporter CKAR. Treatment of COS7 cells expressing CKAR (white) with UTP resulted in a transient increase in endogenous PKC activity, PDBu treatment caused a greater, more sustained induction in PKC activity, and inhibitor treatment resulted in activity returning to baseline (Fig. 6*B*). However, in cells coexpressing CKAR and the PKCα-CDH8 fusion (red), the UTP-mediated increase in PKC activity was reduced. A similar suppression of endogenous activity in response to UTP was observed for the C-deletion protein (yellow), which contains the isolated regulatory moiety of PKC. Although the response to a physiological stimulus (UTP) was significantly suppressed with expression of the regulatory domain fusion, there was no change in PDBu-induced activity. Expression of the C-deletion protein (yellow) suppressed PDBu-induced activity.

Reasoning that the exposed C1 domains of regulatory domain fusion proteins may suppress endogenous PKC activity by sequestering DAG, we examined whether disabling DAG binding abolished the inhibition of UTP-stimulated activity by unmasked regulatory domains. To this end, we introduced a single point mutation in each C1 domain to disrupt DAG binding. Specifically, we mutated W58 and Y123 in the C1A and C1B domains of PKCβII, respectively, to Ala. Coexpression of a catalytically inactive PKC (S660F) (15) with C kinase activity reporter 2 (CKAR2) (62) resulted in suppression of endogenous PKC activity upon UTP stimulation (Fig. 6*C*, red). However, mutation of the C1 domains to disrupt DAG binding abolished suppression of endogenous activity (Fig. 6*C*, blue), resulting in activity similar to that of cells transfected with vector alone (Fig. 6*C*, white). Mutation of these residues also impeded UTP-induced translocation, a consequence of the mutant’s inability to bind DAG (Fig. S5). These data reveal that the dominant-negative effects of proteins with exposed C1 domains specifically result from sequestration of DAG.

To examine the effect of the fusion on basal activity, we treated cells expressing CKAR alone (white) or coexpressing the full-length fusion (red) or C-deletion protein (yellow) with the PKC inhibitor Gӧ6983 (Fig. 6*D*). Expression of either PKCα-CDH8 or the C-deletion variant suppressed endogenous PKC activity, indicated by the smaller reduction in CKAR phosphorylation compared with that of cells expressing the reporter alone. Thus, these results show that the PKCα-CDH8 regulatory domain fusion and C-deletion variant are able to suppress not only agonist-induced PKC activity but also basal PKC activity.

To complement our FRET studies, we also examined the effect of the PKC regulatory domain fusion on endogenous PKC activity by probing for phosphorylation of PKC substrates with a phospho-Ser PKC substrate antibody (Fig. 6*E*). We compared nontransfected (NT) COS7 cells to COS7 cells overexpressing YFP-tagged wild-type PKCα, PKCα-CDH8, or the C-deletion variant in PDBu-treated and vehicle (DMSO) conditions. In the vehicle-treated condition, we detected basal phosphorylation in nontransfected cells that increased with overexpression of wild-type PKCα. Between nontransfected cells and cells expressing the fusion or C-deletion variant, we were unable to detect a difference at basal conditions. In the PDBu-treated condition, we also saw an increase in substrate phosphorylation between the nontransfected cells and cells expressing wild-type PKCα. Importantly, there was significantly less PKC substrate phosphorylation in cells expressing either the regulatory fusion or the C-deletion protein compared with nontransfected cells. These data demonstrate that expression of a PKC regulatory domain fusion (PKCα-CDH8) or the isolated PKC regulatory domain (PKCα-CDH8 C.-Del.) suppresses agonist-evoked phosphorylation of PKC substrates by a mechanism dependent on their ability to bind DAG.

The unmasked regulatory domains of PKC have a propensity to bind the DAG-rich Golgi, leading us to address whether the fusions may have a more pronounced effect in suppressing PKC function at the Golgi (63). Analysis of the mCherry-tagged PKCα-CDH8 fusion in the imaging studies described above revealed that the fusion was more localized to the Golgi compared with wild-type PKCα (Fig. 6*F*). Using the Golgi-targeted FRET reporter GolgiCKAR (64) to monitor PKC activity at the Golgi, overexpression of the PKCα-CDH8 fusion (red) resulted in suppression of both UTP-dependent and PDBu-dependent activation of endogenous PKC (Fig. 6*G*). Addition of the PKC inhibitor Gӧ6983 to assess the effect of the regulatory fusion on basal activity revealed that PKCα-CDH8 expression resulted in almost complete suppression of endogenous PKC activity at the Golgi (Fig. 6*H*). These results corroborate the regulatory domain fusion sequestering DAG at the Golgi, effectively suppressing basal activity of wild-type PKC at this compartment as well as significantly reducing agonist-dependent activation. These data point to the Golgi as an important intracellular compartment in which PKC regulatory domain fusions such as PKCα-CDH8 exert their effects, acting in a dominant-negative manner to suppress global PKC output.

## Discussion

In this study, we show that PKC gene fusions result in proteins that are loss of function in cancer. Specifically, we report that 3' PKC fusions, which retain the gene fragment encoding the PKC catalytic domain, are loss of function because the resulting protein is unstable and targeted for degradation. 5' PKC fusions, which retain the gene fragment encoding the PKC regulatory domain, are not only loss of function as a result of truncation of the catalytic domain but are dominant-negative because the unmasked regulatory domains sequester DAG.

**Figure 7:**
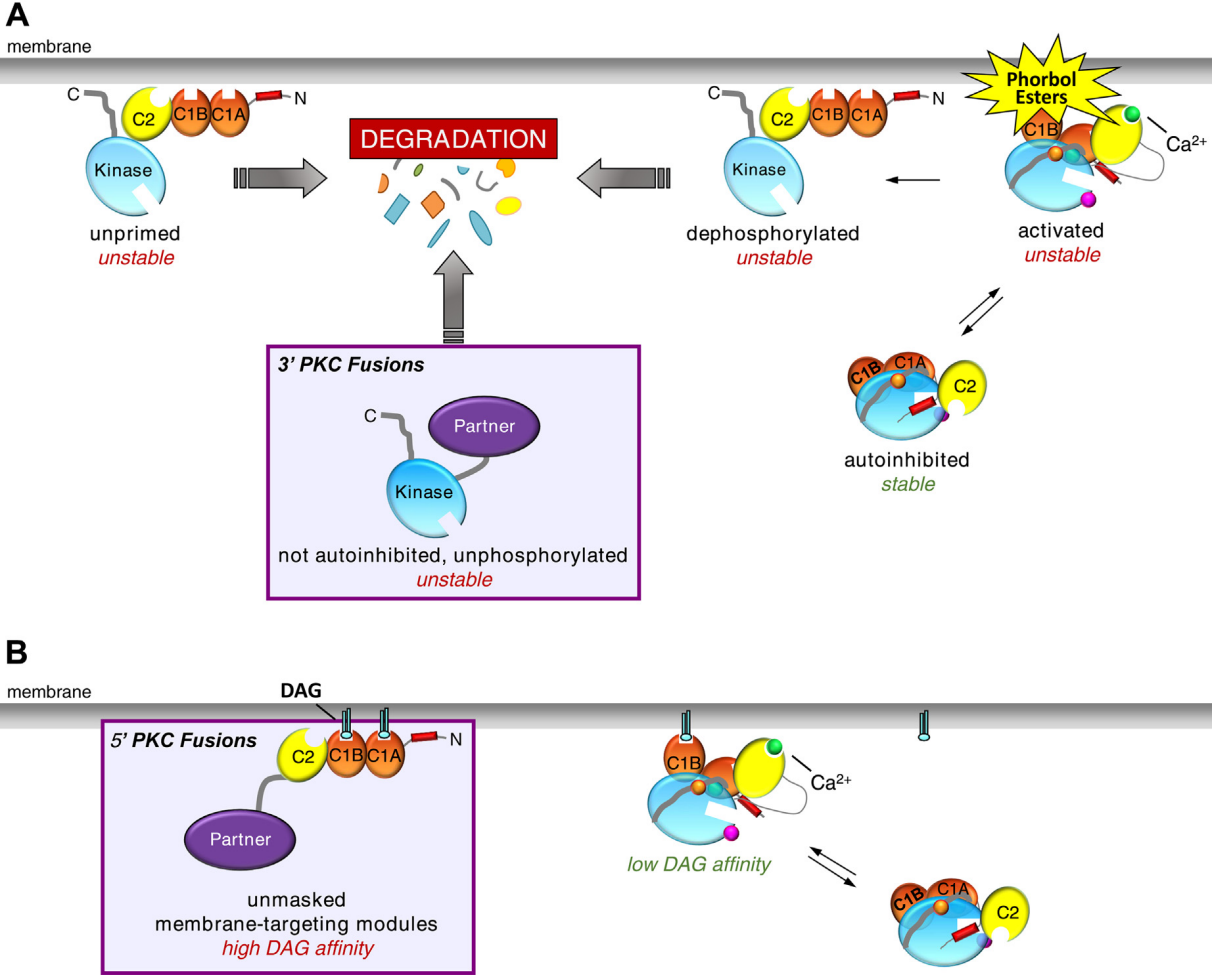
**Model illustrating how PKC fusion proteins are loss of function in cancer.***A*, 3' PKC fusions encode proteins with a constitutively active kinase domain and are unstable. Their inability to autoinhibit renders them sensitive to degradation, preventing accumulation to any detectable level in cells. *B*, 5' PKC fusions encode proteins that lack the kinase domain and are thus intrinsically loss of function but may also act in a dominant-negative manner by sequestering diacylglycerol (DAG).

Advances in bioinformatics and next-generation sequencing technology have revolutionized our ability to appreciate the abundance and relevance of gene fusions in cancer, particularly with respect to solid tumors (38, 65). Whereas numerous studies have investigated point mutations, deletions, and amplifications, relatively little attention has been given to gene fusions. However, as exemplified by *BCR-ABL1* in chronic myeloid leukemia and *ALK* fusions in lung carcinomas and mesenchymal tumors, gene fusions may play critical roles in tumorigenesis and present as attractive targets for therapeutic intervention (18, 66, 67). These well-characterized fusions result in kinases with truncated regulatory domains, resulting in constitutive activity. In addition to *BCR-ABL*, fusions of the kinase domain of PKA in *DNAJB1-PRKACA* and the kinase domain of various FGFR isozymes (*e.g.*, *FGFR3-TACC3*, *BCR-FGFR1*) drive tumorigenesis because of their unregulated activity (68, 69, 70, 71, 72). Our analysis of 3' PKC fusions reveals that these fusions are also constitutively active due to loss of regulatory constraints. However, the PKC catalytic domain fusions are paradoxically loss of function because loss of autoinhibition triggers quality control degradation pathways (Fig. 7*A*).

PKC in an active conformation, with the pseudosubstrate removed from the substrate-binding cavity, is sensitive to dephosphorylation, with the dephosphorylated species subsequently shunted to degradative pathways (22). Thus, aberrant PKC that cannot be autoinhibited is subject to quality control degradation, facilitated by PHLPP (15). PKC that has been activated for too long, which occurs with phorbol ester treatment, is dephosphorylated and also targeted for degradation (50, 51). Overexpression studies revealed that the 3' PKC fusions are capable of being translated into functional, constitutively active protein. However, their inability to retain phosphate in mammalian cells renders them sensitive to degradation. As a result, endogenous catalytic domain fusions are unable to accumulate to detectable levels in cells. Although mRNA of the fusion was detected in CRISPR-edited cells engineered to express the *TANC2-PRKCA* fusion, we were unable to detect the fusion at the protein level. Moreover, treatment of cells with proteasome inhibitors or inhibitors of endosomal/lysosomal degradation and knockdown of known E3 ligases were not sufficient to allow for accumulation of the fusion protein. Identification of the mechanisms causing the degradation of this aberrant PKC will provide potential targets for restoring PKC function in cancers expressing catalytic domain fusions.

Because 5' PKC fusions result in proteins with a truncated catalytic domain, they are intrinsically loss of function with respect to PKC activity. However, we show that these regulatory domain fusions may have an additional role in suppressing wild-type PKC activity. Overexpression of a regulatory domain fusion suppressed endogenous PKC activity in basal and agonist-treated conditions. Furthermore, we identified the Golgi to be a primary site for this fusion-mediated suppression of global PKC output, with the regulatory fusion prelocalized to this intracellular compartment. We have previously shown that the membrane affinity of full-length PKC is at least an order of magnitude less than the product of the affinities of the isolated C1B and C2 domains (73). This reduced affinity of full-length PKC for membranes results from significant masking of the C1 domains during the maturation of PKC to the phosphorylated and autoinhibited species (63). Indeed, in the context of the full-length PKC enzyme, only one of the DAG sensors binds ligand (8, 74), with several lines of evidence suggesting it is the C1B domain (50, 63). Furthermore, not only do the regulatory domain fusions have two unmasked C1 domains but the C1A domain binds DAG with two orders of magnitude higher affinity than the C1B domain (74, 75). As a result, the isolated regulatory domain is predicted to bind DAG with several orders of magnitude higher affinity than full-length PKC. This enhanced affinity for DAG is likely what directs the regulatory domain fusions to the DAG-rich Golgi, overriding the intrinsic affinity of the C2 domain for plasma membrane (76, 77). The PKC regulatory fusions effectively suppressed the basal activity of endogenous PKC, consistent with sequestration of basal DAG, and significantly reduced agonist-evoked activity (Fig. 7*B*). This ability to suppress endogenous activity is consistent with previous studies showing that kinase-dead mutants also act in a dominant-negative manner, suppressing global PKC output (10). Our current results indicate that one mechanism for this dominant-negative effect is mediated by the membrane-targeting modules that are unmasked and can effectively compete for ligand binding.

Functional studies revealed that basal levels of apoptosis are reduced in CRISPR-edited cells expressing the *TANC2-PRKCA* fusion compared with parental cells. This reduction in apoptosis was also observed in CRISPR-edited cells in which one allele of *PRKCA* was deleted. The similar effect on apoptosis resulting from either deletion of *PRKCA* or fusion of *PRKCA* with *TANC2* corroborates loss of PKCα resulting in suppressed apoptosis. These results complement previous studies wherein a heterozygous loss-of-function mutation in PKCβ was corrected to wild-type in DLD-1 colorectal cancer cells through CRISPR/Cas9 gene editing (10). When these cells were subcutaneously injected into nude mice, the resulting tumors were smaller and exhibited greater TUNEL staining compared with those from the parental cells. Supporting a role of conventional PKC isozymes in promoting apoptosis, PKCα has been found to promote apoptosis in LNCaP cells, a model for androgen-dependent prostate cancer, through dephosphorylation and inactivation of Akt (78). It has also been identified as a proapoptotic factor in other cancer contexts, including gastric cancer and leukemia (79, 80, 81). Our findings are thus consistent with expression of the *PRKCA* fusion resulting in increased survival due to loss of one allele of wild-type *PRKCA*.

Although the vast majority of PKC gene fusions are not highly recurrent, they provide an additional general mechanism for loss of PKC function in cancer. The distribution of loss-of-function point mutations in the different domains of PKC revealed that there are no true mutational hotspots, indicating that there are a multitude of ways in which PKC can be inactivated (10). Similarly, although there are many different breakpoints among the PKC fusions, many of the truncation events would result in loss of PKC activity. For 5' PKC fusions, even minimal loss of the C terminus would result in loss of PKC function as phosphorylation of the hydrophobic motif site is necessary for catalytic competence. For 3' PKC fusions, partial loss of the N-terminal regulatory moiety could also result in loss of PKC function as it would constitute a loss in autoinhibition, which could lead to subsequent degradation. Specifically, we showed that deletion of the pseudosubstrate in either PKCα or PKCβII resulted in lack of phosphorylation at the PKC priming sites, agonist-independent cellular activity, and sensitivity to degradation, as exhibited by the fusion proteins. Hence, for conventional isozymes of PKC, any fusion with a minimal truncation of the 5' end of the gene would constitute as loss of function. Overall, due to the potential deletion of functionally relevant domains, PKC gene fusions have a high probability of impacting PKC activity.

Our work underscores the importance of developing therapies that serve to stabilize or enhance PKC activity for cancers in which fusions or mutations of PKC have resulted in loss of PKC function. This is an important consideration as the initial identification of catalytic domain fusions prompted discussion of PKC-targeted inhibitors (24, 34, 82). Our data demonstrate, however, that this would be detrimental. In addition to informing the development of PKC-targeted therapeutics, PKC fusions may also serve another important role in cancer as biomarkers. Recurrent fusions of *PRKCB* and *PRKCD* to membrane-associated proteins were identified in BFHs, one of the most common neoplasms in the skin (23, 24, 26). The recurrent fusions *LAMTOR1-PRKCD* and *PDPN-PRKCB*, alongside *CD63-PRKCD* and *KIRREL-PRKCA*, were identified through fluorescent *in situ* hybridization (FISH) and RT-qPCR. In each of these cases, the N terminus of PKC was replaced with membrane-binding domains of a partner protein while retaining the PKC catalytic domain. In total, roughly 25% of BFHs analyzed contained a PKC fusion. Moreover, tumors containing rearrangements in *PRKCB* did not have rearrangements in *ALK*, previously shown to occur in the majority of the epithelial subtype of BFHs. The *SLC44A1-PRKCA* fusion, characterized in this study, was identified in rare mixed neuronal-glial tumors known as PGNTs that are difficult to diagnose (35). In the initial analyses of these tumors, the sole karyotypic abnormality was the chromosomal translocation t(9;17)(q31;q24), resulting in the *SLC44A1-PRKCA* fusion. Out of the ten documented cases of PGNTs, the fusion was detected in nine of them (35, 36, 37). Furthermore, PGNTs harboring the PKC fusion lacked *BRAF* and *FGFR1* mutations characteristic of histologically similar tumors. Interestingly, the fusion was not detected in any of 15 PGNT “mimics,” tumors that shared many morphological similarities to *bona fide* PGNTs (37). Taken together, the recurrent PKC fusions detected in BFHs and PGNTs and mutations and rearrangements in known drivers appear to be mutually exclusive, so these PKC fusions may serve as effective biomarkers. Thus, PKC gene fusions, whether as targets or biomarkers, may have significant therapeutic relevance in cancer.

## Experimental procedures

### Curation of PKC gene fusions

5' and 3' PKC fusions organized in Table S1, Table S2, and graphed in Figure 1, *B* and *C* were curated from the literature (18, 23, 24, 25, 26, 27, 28, 29, 30, 31, 32, 33, 34, 35, 36, 37) and the following online databases: the Mitelman Database of Chromosome Aberrations and Gene Fusions in Cancer (mitelmandatabase.isb-cgc.org), the TCGA Tumor Fusion Gene Data Portal, ChimerDB v3.0, and COSMIC (cancer.sanger.ac.uk) (30, 38, 39, 40). Fusions were detected in the following cancer types: BFH, bladder urothelial carcinoma, BRCA, cervical squamous cell carcinoma and endocervical adenocarcinoma, colon adenocarcinoma, lymphoid neoplasm diffuse large B-cell lymphoma, esophageal carcinoma, glioblastoma multiforme, head and neck squamous cell carcinoma, clear cell renal cell carcinoma, kidney renal papillary cell carcinoma, acute myeloid leukemia, LGG, liver hepatocellular carcinoma, LUAD, LUSC, medulloblastoma, ovarian serous cystadenocarcinoma, pheochromocytoma and paraganglioma, PGNT, prostate adenocarcinoma, sarcoma, skin cutaneous melanoma, stomach adenocarcinoma, T-cell acute lymphoblastic leukemia, and UCEC.

### Plasmid constructs

The CKAR, CKAR2, and the Golgi-targeted C Kinase Activity Reporter (GolgiCKAR) were previously described (46, 62, 64). Human PKCα and PKCβII were N-terminally tagged with mCherry and YFP *via* Gateway cloning as previously described (10). PKC pseudosubstrate-deleted constructs (ΔPS) were generated by QuikChange site-directed mutagenesis (Agilent) as previously described (15). Human TANC2-PKCα and GGA2-PKCβII constructs were generated using Gibson Assembly (NEB). Constructs containing human CTL1 (*SLC44A1*) and CDH8 were obtained through the DNASU plasmid repository (HsCD00627280 and HsCD00403546, respectively). The CTL1-PKCα and PKCα-CDH8 fusions were generated through multiple iterations of PCR. Fragments of each protein were amplified by PCR with primers containing overhang homologous to their fusion partner. Once the fragments were annealed, the complete fusion was amplified from the reaction by PCR and subcloned into pcDNA3. N-deletion and C-deletion constructs were generated through amplification of regions of interest by PCR from constructs of wild-type PKCα and subsequent subcloning into pcDNA3. Constructs with an N-terminal mCherry tag were subcloned into pcDNA3 in which mCherry had been subcloned, and constructs with an N-terminal YFP tag were subcloned into pcDNA3 in which YFP had been subcloned. Point mutants were generated using QuikChange site-directed mutagenesis. The PX458 vector encoding the Cas9 nuclease was a gift from Feng Zhang (Plasmid # 48,138, Addgene). The pAAV-SEPT-Acceptor vector was a gift from Todd Waldman (Plasmid #25648, Addgene).

### Antibodies and reagents

The antibodies used in this study are as listed: PKCα (610108, BD Transduction), PKCβII (610128, BD Transduction), phospho-specific PKC turn motif (PKCα/β pT638/641, 9375S, Cell Signaling), phospho-specific PKC hydrophobic motif (PKCβ pS660, 9371S, Cell Signaling), HA (clone 16B12, 901515, BioLegend), Hsp90 (610418, BD Transduction), TANC2 (D-11, sc-515710, Santa Cruz), caspase-3 (9662S, Cell Signaling), cleaved caspase-3 (Asp175, 9661S, Cell Signaling), pSer PKC Substrate (2261S, Cell Signaling), GFP (2555S, Cell Signaling), β-actin (A2228, Sigma-Aldrich), and α-tubulin (T6074, Sigma-Aldrich). The phospho-specific PKC activation loop antibody has been previously described (83). The pharmacological reagents used in this study are as listed: UTP (6701, Calbiochem), PDBu (524390, Calbiochem), Gӧ6976 (365253, Calbiochem), Gӧ6983 (80,051–928, Calbiochem), BisIV (203297, Calbiochem), and etoposide (341205, Calbiochem).

### Cell culture and transfection

All cells were maintained in Dulbecco’s modified Eagle’s medium (DMEM, 10–013-CV, Corning) containing 10% fetal bovine serum (S11150, Atlanta Biologicals) and 1% penicillin/streptomycin (15,140–122, Gibco) at 37 °C in 5% CO_2_ (v/v). Cells were periodically tested for *Mycoplasma* contamination by a PCR-based method and showed no contamination (84). Transient transfection was carried out using the Lipofectamine 3000 transfection reagent (Life Technologies), as indicated by the manufacturer’s instructions.

### Cell lysis and immunoblotting

Most cells were lysed in buffer containing 50 mM Tris (pH 7.4), 1% Triton X-100, 50 mM NaF, 10 mM Na_4_P_2_O_7_, 100 mM NaCl, 5 mM EDTA, 1% deoxycholic acid that was supplemented with 1 mM Na_3_VO_4_, 1 mM phenylmethyl-sulfonyl fluoride, 50 μg/ml leupeptin, 1 μM microcystin, 1 mM DTT, and 2 mM benzamidine. Whole-cell lysates were briefly sonicated and boiled in sample buffer containing 250 mM Tris HCl, 8% (w/v) SDS, 40% (v/v) glycerol, 80 μg/ml bromophenol blue, and 2.86 M β-mercaptoethanol for 5 min at 95 °C. Cells used in the analysis of the CTL1-PKCα fusion protein were lysed in RIPA buffer (50 mM Tris (pH 7.4), 150 mM NaCl, 2 mM EDTA, 1% Triton X-100, 1% deoxycholic acid, 0.1% SDS, 10 mM NaF) supplemented with 1 mM Na_3_VO_4_, 1 mM phenylmethyl-sulfonyl fluoride, 50 μg/ml leupeptin, 1 μM microcystin, 1 mM DTT, and 2 mM benzamidine. Whole-cell lysates from these studies were neither sonicated nor boiled to minimize protein aggregation. Protein was quantified by Bradford assay (5000006, Bio-Rad) (85). Lysates were analyzed by SDS-PAGE and immunoblotting *via* chemiluminescence on a FluorChem Q imaging system (ProteinSimple).

### FRET imaging and analysis

Cells were imaged as previously described (64). As indicated, COS7 cells were transfected with CKAR, CKAR2, or GolgiCKAR alone or coexpressed with mCherry-tagged PKC. Cells were rinsed once with and imaged in Hanks’ balanced salt solution (Corning) containing 1 mM CaCl_2_. Images were acquired on a Zeiss Axiovert microscope (Carl Zeiss MicroImaging, Inc) using a MicroMax digital camera (Roper-Princeton Instruments) controlled by MetaFluor software (Universal Imaging Corp.). Baseline values were acquired for at least 2 min prior to reagent addition. For experiments measuring agonist-induced activity, data were normalized to FRET ratios after addition of inhibitor. For experiments measuring basal activity, data were normalized to FRET ratios at baseline. Normalized FRET ratios from at least three independent experiments were combined, and traces represent the average of these ratios ± SEM.

### Cellular ubiquitination assay

COS7 cells were transfected with HA-tagged ubiquitin alone or cotransfected with the indicated YFP-tagged PKC. After 24 h, cells were lysed in 50 mM Tris (pH 7.4), 1% Triton X-100, 50 mM NaF, 10 mM Na_4_P_2_O_7_, 100 mM NaCl, 5 mM EDTA, 1% deoxycholic acid that was supplemented with 1 mM Na_3_VO_4_, 1 mM phenylmethyl-sulfonyl fluoride, 50 μg/ml leupeptin, 1 μM microcystin, 1 mM DTT, and 2 mM benzamidine. Lysis buffer was also supplemented with 10 mM N-ethylmaleimide to preserve ubiquitinated species. After centrifugation, 500 μg of the detergent-solubilized lysates was incubated with an anti-PKCα antibody overnight at 4 °C and subsequently with protein A/G beads (Santa Cruz) for 1 h at 4 °C. The immunocomplexes were washed three times with lysis buffer (unsupplemented) and eluted by boiling in sample buffer for 5 min at 95 °C. Samples were separated by SDS-PAGE and analyzed by immunoblotting.

### Generation of CRISPR constructs for *TANC2-PRKCA* expression

*TANC2-PRKCA* is generated from the fusion of exons 1–2 of *TANC2* to exons 6–17 of *PRKCA*. To generate the *TANC2-PRKCA* gene fusion, sgRNAs targeting intron 2 of *TANC2* (*TANC2* gRNA: GTTGGAGTATCATCACA-CAC) and intron 6 of *PRKCA* (*PRKCA* gRNA: GACCGGAATTCCCTATCCAG) were cloned into the PX458 vector. Left and right homology arms to target *TANC2* and *PRKCA*, respectively, were approximately 1 kb each and generated using the following oligonucleotides:

***TANC2*** **LHA**

forward: GTTTTAGCTTTTCTTAACATGCATGAGGG

reverse: CAAATGTTGGTGTTGAAAATTCACTTTTGG

***PRKCA*** **RHA**

forward: GGGCCTGGTTACTCCCTCCTCGGGGCTG

reverse: CTCGGCTGCAAAATCTGCTCCCCCCATACATGG

Homology arms were cloned into the pAAV-SEPT-Acceptor vector.

### Generation of rAAV for *TANC2-PRKCA* expression

Recombinant AAV (rAAV) particles were generated in HEK 293T cells (ATCC CRL-3216) *via* cotransfection of pAAV-SEPT containing the *TANC2* and *PRKCA* homology arms with the packaging plasmids pAAV-RC and pHELPER. Five days after transfection, cells were collected in media and subjected to three freeze/thaw cycles to release rAAV particles followed by a 15 min spin to remove cellular debris. Released and clarified rAAV particles were stored in media at –80 °C.

### Generation of CRISPR-edited cell lines for *TANC2-PRKCA* expression

MDA-MB-231 cells at 50–60% confluence in a 10 cm dish were placed in serum-free media and transfected with 6 μg each of PX458-*TANC2* sgRNA and PX458-*PRKCA* sgRNA using Lipofectamine 3000. After 4 h, cells were fed with complete media and incubated for 2 h. To transduce the cells with rAAV, media was replaced with 50/50 media/rAAV and incubated for 2 days. To select recombinant clones, cells were trypsinized and collected in 120 ml of media containing 2 mg/ml G418 and seeded at 200 μl/well into six 96-well plates. Plates were wrapped in plastic wrap to reduce evaporation and incubated for 20–30 days. Single colonies were expanded into 48-well plates and screened for generation of the fusion product by PCR. To screen for recombinant clones, gDNA was isolated using the *Quick*-DNA Microprep Kit (D3020, Zymo Research) from 90% of the cells with the remaining 10% reseeded in a 48-well plate. For clones that successfully integrated the repair template, the floxed Neo cassette was removed. To remove the cassette from the clonal cell lines, cells were grown in a 6-well plate to 50% confluence and treated with Adenovirus-Cre (Ad5CMVCre-eGFP high titer, Iowa U.) in serum-free media overnight before replacing with complete medium. Efficiency of cassette removal was verified by splitting the well into two in the presence and absence of 2 mg/ml G418.

### mRNA isolation and RT-qPCR analysis

RNA was isolated from cells using the RNeasy Mini Kit (74104, Qiagen). Total mRNA was reverse-transcribed into cDNA using the SuperScript III First Strand Synthesis System (18080-051, Invitrogen), and 20 ng of the resulting cDNA was used to perform real-time quantitative PCR. Real-time quantitative PCR was performed with SYBR Premix Ex TaqII (RR820A, Takara Bio) and a 100 nM mix of forward and reverse primers on a QuantStudio 3 Real-Time PCR System (Applied Biosystems). The real-time quantitative PCR values were normalized to the housekeeping gene actin (*ACTB*) using the ΔΔCT method (86). The primer sequences used in this study are as follows:

**human *PRKCA* 5'**

forward: CGGCGGAGGCAAGAGGTGGT

reverse: TGCGGGCGAAGCGGTTG

**human *PRKCA* 3'**

forward: ATTCAAGCCCAAAGTGTGTGG

reverse: GATCAGGTGGTGTTAAGACGGG

**human *TANC2* 5'**

forward: CAGGTCAAGTGATGGAGGG

reverse: CAGAGCGGCTTTGGCGAG

**human** ***TANC2 3'***

forward: GCTTTGCACCTTATAGGCCTCC

reverse: GGAAAACCCAATCTCGGCCAAC

**human** ***TANC2-PRKCA***

forward: GAGGAACCACCGGATCGAAG

reverse: GGATCTGAAAGCCCGTTTGG

**human** ***ACTB***

forward: AGGCACCAGGGCGTGAT

reverse: GCCCACATAGGAATCCTTCTGAC

## Generation of CRISPR-edited cell lines for knockout of PKCα

To generate a PKCα knockout line, a gRNA was selected using Synthego’s CRISPR Design Tool. A sgRNA targeting exon 4 of *PRKCA* was cloned into the PX458 vector (*PRKCA* gRNA 3: AGGTGGGGCTTCCGTAAGTG). MDA-MB-231 cells were nucleofected with 2 μg of the sgRNA using the Amaxa Nucleofector II (Amaxa) and incubated for 3 days. Cells were then single-cell sorted for GFP-positive cells into two 96-well plates and incubated for 20–30 days. Single colonies were expanded into 48-well plates and screened for potential *PRKCA* knockout by PCR. To screen clones, gDNA was isolated using the *Quick*-DNA Microprep Kit (D3020, Zymo Research) from 90% of the cells with the remaining 10% reseeded in a 48-well plate. An approximately 1 kb fragment around the targeting region of the sgRNA was amplified by PCR and submitted for Sanger sequencing (GENEWIZ). The results were analyzed using Synthego’s Inference of CRISPR Edits (ICE) CRISPR Analysis Tool and validated by immunoblotting following sufficient expansion of the clones.

## Apoptosis assay

MDA-MB-231 cells were seeded at a density of 10,000 cells/well in a 96-well plate 24 h before performing the assay. Apoptosis was measured using the RealTime-Glo Annexin V Apoptosis and Necrosis Assay (Promega, JA1011), which is based on luminescence upon binding of annexin V-luciferase to cell-surface-exposed phosphatidylserine in real time. Luminescence was measured at the indicated times after addition of vehicle (DMSO) or etoposide (12.5 μM) on a Tecan SPARK multiwell plate reader, following manufacturer’s instructions. Data represent the mean ± SEM from three independent experiments, and statistical significance was determined using a Student’s *t-*test.

## Data availability

All data are contained within the article.

## Supporting information

This article contains supporting information (57, 87).

## Conflict of interest

The authors declare that they have no conflicts of interest with the contents of this article.

## References

[1] Dempsey E.C., Newton A.C., Mochly-rosen D., Fields A.P., Reyland M.E., Insel P.A., Messing R.O., Edward C., Mochly D. (2000). Protein kinase C isozymes and the regulation of diverse cell responses. Am. J. Physiol. Lung Cell. Mol. Physiol..

[2] Garg R., Benedetti L.G., Abera M.B., Wang H., Abba M., Kazanietz M.G. (2014). Protein kinase C and cancer: What we know and what we do not. Oncogene.

[3] Griner E.M., Kazanietz M.G. (2007). Protein kinase C and other diacylglycerol effectors in cancer. Nat. Rev. Cancer.

[4] Noh H., King G.L. (2007). The role of protein kinase C activation in diabetic nephropathy. Kidney Int..

[5] Callender J.A., Newton A.C. (2017). Conventional protein kinase C in the brain: 40 years later. Neuronal Signal.

[6] Newton A.C., Brognard J. (2017). Reversing the Paradigm: Protein kinase C as a tumor suppressor. Trends Pharmacol. Sci..

[7] Castagnag M., Yoshimi T., Kaibuchi K., Sano K., Kikkawa U., Nishizuka Y. (1982). Direct activation of calcium-activated, phospholipid-dependent protein kinase by tumor-promoting phorbol esters. J. Biol. Chem..

[8] Kikkawas U., Takai Y., Tanaka Y., Miyake R., Nishizuka Y. (1983). Protein kinase C as a possible receptor protein of tumor-promoting phorbol esters. J. Biol. Chem..

[9] Leach K.L., James M.L., Blumberg P.M. (1983). Characterization of a specific phorbol ester aporeceptor in mouse brain cytosol. Proc. Natl. Acad. Sci. U. S. A..

[10] Antal C.E., Hudson A.M., Kang E., Zanca C., Wirth C., Stephenson N.L., Trotter E.W., Gallegos L.L., Miller C.J., Furnari F.B., Hunter T., Brognard J., Newton A.C. (2015). Cancer-associated protein kinase C mutations reveal kinase’s role as tumor suppressor. Cell.

[11] Kuehn H.S., Niemela J.E., Rangel-Santos A., Zhang M., Pittaluga S., Stoddard J.L., Hussey A.A., Evbuomwan M.O., Priel D.A.L., Kuhns D.B., Park C.L., Fleisher T.A., Uzel G., Oliveira J.B. (2013). Loss-of-function of the protein kinase C δ (PKCδ) causes a B-cell lymphoproliferative syndrome in humans. Blood.

[12] Salzer E., Santos-Valente E., Klaver S., Ban S.A., Emminger W., Prengemann N.K., Garncarz W., Müllauer L., Kain R., Boztug H., Heitger A., Arbeiter K., Eitelberger F., Seidel M.G., Holter W. (2013). B-cell deficiency and severe autoimmunity caused by deficiency of protein kinase C δ. Blood.

[13] Belot A., Kasher P.R., Trotter E.W., Foray A.P., Debaud A.L., Rice G.I., Szynkiewicz M., Zabot M.T., Rouvet I., Bhaskar S.S., Daly S.B., Dickerson J.E., Mayer J., O’Sullivan J., Juillard L. (2013). Protein kinase Cδ deficiency causes mendelian systemic lupus erythematosus with B cell-defective apoptosis and hyperproliferation. Arthritis Rheum..

[14] Dowling C.M., Phelan J., Callender J.A., Cathcart M.C., Mehigan B., McCormick P., Dalton T., Coffey J.C., Newton A.C., O’Sullivan J., Kiely P.A. (2016). Protein kinase C beta II suppresses colorectal cancer by regulating IGF-1 mediated cell survival. Oncotarget.

[15] Baffi T.R., Van A.A.N., Zhao W., Mills G.B., Newton A.C. (2019). Protein kinase C quality control by phosphatase PHLPP1 Unveils loss-of-function mechanism in cancer. Mol. Cell.

[16] Halvorsen A.R., Haugen M.H., Öjlert Å.K., Lund-Iversen M., Jørgensen L., Solberg S., Mælandsmo G.M., Brustugun O.T., Helland Å. (2020). Protein kinase C isozymes associated with Relapse free survival in non-small cell lung cancer patients. Front. Oncol..

[17] Tovell H., Newton A.C. (2021). PHLPPing the balance: Restoration of protein kinase C in cancer. Biochem. J..

[18] Yoshihara K., Wang Q., Torres-Garcia W., Zheng S., Vegesna R., Kim H., Verhaak R.G.W. (2015). The landscape and therapeutic relevance of cancer-associated transcript fusions. Oncogene.

[19] Newton A.C. (2010). Protein kinase C: Poised to signal. Am. J. Physiol. Endocrinol. Metab..

[20] Tobias I.S., Newton A.C. (2016). Protein scaffolds control localized protein kinase Cζ activity. J. Biol. Chem..

[21] Keranen L.M., Dutil E.M., Newton A.C. (1995). Protein kinase C is regulated *in vivo* by three functionally distinct phosphorylations. Curr. Biol..

[22] Gao T., Brognard J., Newton A.C. (2008). The phosphatase PHLPP controls the cellular levels of protein kinase C. J. Biol. Chem..

[23] Walther C., Hofvander J., Nilsson J., Magnusson L., Domanski H.A., Gisselsson D., Tayebwa J., Doyle L.A., Fletcher C.D.M., Mertens F. (2015). Gene fusion detection in formalin-fixed paraffin-embedded benign fibrous histiocytomas using fluorescence *in situ* hybridization and RNA sequencing. Lab. Investig..

[24] Płaszczyca A., Nilsson J., Magnusson L., Brosjö O., Larsson O., Vult Von Steyern F., Domanski H.A., Lilljebjörn H., Fioretos T., Tayebwa J., Mandahl N., Nord K.H., Mertens F. (2014). Fusions involving protein kinase C and membrane-associated proteins in benign fibrous histiocytoma. Int. J. Biochem. Cell Biol..

[25] Luo J., Gu D., Lu H., Liu S., Kong J. (2019). Coexistence of a novel PRKCB-ALK , EML4-ALK Double-fusion in a lung adenocarcinoma patient and response to Crizotinib. J. Thorac. Oncol..

[26] Panagopoulos I., Gorunova L., Bjerkehagen B., Lobmaier I., Heim S. (2015). LAMTOR1-PRKCD and NUMA1-SFMBT1 fusion genes identified by RNA sequencing in aneurysmal benign fibrous histiocytoma with t(3;11)(p21;q13). Cancer Genet..

[27] Robinson D.R., Wu Y., Lonigro R.J., Vats P., Cobain E., Everett J., Cao X., Rabban E., Kumar-sinha C., Raymond V., Schuetze S., Alva A., Siddiqui J., Chugh R., Worden F. (2017). Integrative clinical genomics of metastatic cancer. Nature.

[28] Liu Y., Easton J., Shao Y., Maciaszek J., Wang Z., Wilkinson M.R., Mccastlain K., Edmonson M., Pounds S.B., Shi L., Zhou X., Ma X., Sioson E., Li Y., Rusch M. (2017). The genomic landscape of pediatric and young adult T-lineage acute lymphoblastic leukemia. Nat. Genet..

[29] Dupain C., Harttrampf A.C., Boursin Y., Lebeurrier M., Rondof W., Robert-siegwald G., Khoueiry P., Geoerger B., Massaad-massade L. (2019). Discovery of New fusion transcripts in a Cohort of pediatric solid cancers at Relapse and relevance for Personalized Medicine. Mol. Ther..

[30] Hu X., Wang Q., Tang M., Barthel F., Amin S., Yoshihara K., Lang F.M., Martinez-ledesma E., Lee S.H., Zheng S., Verhaak R.G.W. (2017). TumorFusions : An integrative resource for cancer-associated transcript fusions. Nucleic Acids Res..

[31] Gao Q., Liang W., Foltz S.M., Nykter M., Gao Q., Liang W., Foltz S.M., Mutharasu G., Jayasinghe R.G. (2018). Driver Fusions and Their Implications in the Development and Treatment of Human Cancers Resource Driver Fusions and Their Implications in the Development and Treatment of Human Cancers. Cell Rep..

[32] Matissek K.J., Onozato M.L., Sun S., Zheng Z., Schultz A., Lee J., Patel K., Jerevall P., Saladi S.V., Macleay A., Tavallai M., Badovinac-crnjevic T., Barrios C., Beşe N., Chan A. (2018). Expressed gene fusions as frequent drivers of poor Outcomes in Hormone receptor – positive breast cancer. Cancer Discov..

[33] Kim J., Kim S., Ko S., In Y., Moon H., Ahn S.K., Kim M.K. (2015). Recurrent fusion transcripts detected by Whole-transcriptome sequencing of 120 primary breast cancer samples. Genes. Chromosomes Cancer.

[34] Stransky N., Cerami E., Schalm S., Kim J.L., Lengauer C. (2014). The landscape of kinase fusions in cancer. Nat. Commun..

[35] Bridge J.A., Liu X.Q., Sumegi J., Nelson M., Reyes C., Bruch L.A., Rosenblum M., Puccioni M.J., Bowdino B.S., McComb R.D. (2013). Identification of a novel, recurrent SLC44A1-PRKCA fusion in papillary glioneuronal tumor. Brain Pathol..

[36] Nagaishi M., Nobusawa S., Matsumura N., Kono F., Ishiuchi S., Abe T., Ebato M., Wang Y., Hyodo A., Yokoo H., Nakazato Y. (2016). SLC44A1-PRKCA fusion in papillary and rosette-forming glioneuronal tumors. J. Clin. Neurosci..

[37] Pages M., Lacroix L., Tauziede-Espariat A., Castel D., Daudigeos-Dubus E., Ridola V., Gilles S., Fina F., Andreiuolo F., Polivka M., Lechapt-Zalcman E., Puget S., Boddaert N., Liu X.Q., Bridge J.A. (2015). Papillary glioneuronal tumors: Histological and molecular characteristics and diagnostic value of SLC44A1-PRKCA fusion. Acta Neuropathol. Commun..

[38] Mitelman F., Johansson B., Mertens F. (2007). The impact of translocations and gene fusions on cancer causation. Nat. Rev. Cancer.

[39] Lee M., Lee K., Yu N., Jang I., Choi I., Kim P., EunJang Y., Kim B., Kim S., Lee B., Kang J., Lee S. (2017). ChimerDB 3.0: An enhanced database for fusion genes from cancer transcriptome and literature data mining. Nucleic Acids Res..

[40] Tate J.G., Bamford S., Jubb H.C., Sondka Z., Beare D.M., Bindal N., Boutselakis H., Cole C.G., Creatore C., Dawson E., Fish P., Harsha B., Hathaway C., Jupe S.C., Kok Y. (2019). Cosmic: The Catalogue of Somatic mutations in cancer. Nucleic Acids Res..

[41] Kim R.N., Moon H.G., Han W., Noh D.Y. (2018). Perspective insight into future potential fusion gene transcript biomarker candidates in breast cancer. Int. J. Mol. Sci..

[42] Gasparini A., Tosatto S.C.E., Murgia A., Leonardi E. (2017). Dynamic scaffolds for neuronal signaling: In silico analysis of the TANC protein family. Sci. Rep..

[43] Han S., Nam J., Li Y., Kim S., Cho S.H., Cho Y.S., Choi S.Y., Choi J., Han K., Kim Y., Na M., Kim H., Bae Y.C., Choi S.Y., Kim E. (2010). Regulation of dendritic spines, spatial memory, and embryonic development by the TANC family of PSD-95-interacting proteins. J. Neurosci..

[44] Hedtke V., Bakovic M. (2019). Choline transport for phospholipid synthesis: An emerging role of choline transporter-like protein 1. Exp. Biol. Med..

[45] Von Einem B., Wahler A., Schips T., Serrano-Pozo A., Proepper C., Boeckers T.M., Rueck A., Wirth T., Hyman B.T., Danzer K.M., Thal D.R., Von Arnim C.A.F. (2015). The golgi-localized γ-ear-containing ARF-binding (GGA) proteins alter amyloid-β precursor protein (APP) processing through interaction of their GAE domain with the beta-site APP cleaving enzyme 1 (BACE1). PLoS One.

[46] Violin J.D., Zhang J., Tsien R.Y., Newton A.C. (2003). A genetically encoded fluorescent reporter reveals oscillatory phosphorylation by protein kinase C. J. Cell Biol..

[47] Taylor S.S., Kornev A.P. (2011). Protein kinases: Evolution of dynamic regulatory proteins. Trends Biochem. Sci..

[48] Hu J., Ahuja L.G., Meharena H.S., Kannan N., Kornev A.P., Taylor S.S., Shaw A.S. (2015). Kinase regulation by hydrophobic spine Assembly in cancer. Mol. Cell. Biol..

[49] Behn-Krappa A., Newton A.C. (1999). The hydrophobic phosphorylation motif of conventional protein kinase C is regulated by autophosphorylation. Curr. Biol..

[50] Szallasi Z., Smith C.B., Pettit G.R., Blumberg P.M. (1994). Differential regulation of protein kinase C isozymes by bryostatin 1 and phorbol 12-myristate 13-acetate in NIH 3T3 fibroblasts. J. Biol. Chem..

[51] Kraft A.S., Anderson W.B., Cooper H.L., Sando J.J. (1982). Decrease in cytosolic calcium/phospholipid-dependent protein kinase activity following phorbol ester treatment of EL4 thymoma cells. J. Biol. Chem..

[52] Gould C.M., Kannan N., Taylor S.S., Newton A.C. (2009). The chaperones Hsp90 and Cdc37 mediate the maturation and stabilization of protein kinase C through a conserved PXXP motif in the C-terminal tail. J. Biol. Chem..

[53] Biebl M.M., Buchner J. (2019). Structure, function, and regulation of the hsp90 machinery. Cold Spring Harb. Perspect. Biol..

[54] Sima S., Richter K. (2018). Regulation of the Hsp90 system. Biochim. Biophys. Acta - Mol. Cell Res..

[55] Kaulich M., Lee Y.J., Lönn P., Springer A.D., Meade B.R., Dowdy S.F. (2015). Efficient CRISPR-rAAV engineering of endogenous genes to study protein function by allele-specific RNAi. Nucleic Acids Res..

[56] Kaulich M., Dowdy S.F. (2015). Combining CRISPR/Cas9 and rAAV templates for Efficient gene editing. Nucleic Acid Ther..

[57] Chen D., Gould C., Garza R., Gao T., Hampton R.Y., Newton A.C. (2007). Amplitude control of protein kinase C by RINCK, a novel E3 ubiquitin ligase. J. Biol. Chem..

[58] Murata S., Minami Y., Minami M., Chiba T., Tanaka K. (2001). CHIP is a chaperone-dependent E3 ligase that ubiquitylates unfolded protein. EMBO Rep..

[59] Porter A.G., Jänicke R.U. (1999). Emerging roles of caspase-3 in apoptosis. Cell Death Differ..

[60] Thornberry N.A., Lazebnik Y. (1998). Caspases: Enemies within. Science (80-).

[61] Memi F., Killen A.C., Barber M., Parnavelas J.G., Andrews W.D. (2019). Cadherin 8 regulates proliferation of cortical interneuron progenitors. Brain Struct. Funct..

[62] Ross B.L., Tenner B., Markwardt M.L., Zviman A., Shi G., Kerr J.P., Snell N.E., Mcfarland J.J., Mauban J.R., Ward C.W., Rizzo M.A., Zhang J. (2018). Single-color , ratiometric biosensors for detecting signaling activities in live cells. eLife.

[63] Antal C.E., Violin J.D., Kunkel M.T., Skovsø S., Newton A.C. (2014). Intramolecular conformational changes optimize protein kinase C signaling. Chem. Biol..

[64] Gallegos L.L., Kunkel M.T., Newton A.C. (2006). Targeting protein kinase C activity reporter to discrete intracellular regions reveals spatiotemporal differences in agonist-dependent signaling. J. Biol. Chem..

[65] Mertens F., Johansson B., Fioretos T., Mitelman F. (2015). The emerging complexity of gene fusions in cancer. Nat. Rev. Cancer.

[66] Seo J.S., Ju Y.S., Lee W.C., Shin J.Y., Lee J.K., Bleazard T., Lee J., Jung Y.J., Kim J.O., Shin J.Y., Yu S.B., Kim J., Lee E.R., Kang C.H., Park I.K. (2012). The transcriptional landscape and mutational profile of lung adenocarcinoma. Genome Res..

[67] Hantschel O. (2012). Structure, regulation, signaling, and targeting of Abl kinases in cancer. Genes and Cancer.

[68] Tomasini M.D., Wang Y., Karamafrooz A., Li G., Beuming T., Gao J., Taylor S.S., Veglia G., Simon S.M. (2018). Conformational landscape of the PRKACA-DNAJB1 Chimeric kinase, the driver for fibrolamellar hepatocellular carcinoma. Sci. Rep..

[69] Kastenhuber E.R., Lalazar G., Houlihan S.L., Tschaharganeh D.F., Baslan T., Chen C.C., Requena D., Tian S., Bosbach B., Wilkinson J.E., Simon S.M., Lowe S.W. (2017). DNAJB1–PRKACA fusion kinase interacts with β-catenin and the liver regenerative response to drive fibrolamellar hepatocellular carcinoma. Proc. Natl. Acad. Sci. U. S. A..

[70] Nelson K.N., Meyer A.N., Wang C.G., Donoghue D.J. (2018). Oncogenic driver FGFR3-TACC3 is dependent on membrane trafficking and ERK signaling. Oncotarget.

[71] Peiris M.N., Meyer A.N., Nelson K.N., Bisom-Rapp E.W., Donoghue D.J. (2020). Oncogenic fusion protein BCR-FGFR1 requires the breakpoint cluster region-mediated oligomerization and chaperonin Hsp90 for activation. Haematologica.

[72] Li F., Peiris M.N., Donoghue D.J. (2020). Functions of FGFR2 corrupted by translocations in intrahepatic cholangiocarcinoma. Cytokine Growth Factor Rev..

[73] Johnson J.E., Giorgione J., Newton A.C. (2000). The C1 and C2 domains of protein kinase C are independent membrane targeting modules, with specificity for phosphatidylserine conferred by the C1 domain. Biochemistry.

[74] Giorgione J., Hysell M., Harvey D.F., Newton A.C. (2003). Contribution of the C1A and C1B domains to the membrane interaction of protein kinase C. Biochemistry.

[75] Dries D.R., Gallegos L.L., Newton A.C. (2007). A single residue in the C1 domain sensitizes novel protein kinase C isoforms to cellular diacylglycerol production. J. Biol. Chem..

[76] Verdaguer N., Corbalan-garcia S., Ochoa W.F., Fita I., Go J.C. (1999). Verdaguer-1999-Ca(2+) bridges the C2 membrane-. EMBO J.

[77] Guerrero-Valero M., Ferrer-Orta C., Querol-Audí J., Marin-Vicente C., Fita I., Gómez-Fernández J.C., Verdaguer N., Corbalán-García S. (2009). Structural and mechanistic insights into the association of PKCα-C2 domain to PtdIns(4,5)P 2. Proc. Natl. Acad. Sci. U. S. A..

[78] Tanaka Y., Gavrielides M.V., Mitsuuchi Y., Fujii T., Kazanietz M.G. (2003). Protein kinase C promotes apoptosis in LNCaP prostate cancer cells through activation of p38 MAPK and inhibition of the Akt survival pathway. J. Biol. Chem..

[79] Nakashima S. (2002). JB Minireview-protein kinase C Isotypes and their specific function protein kinase Shigeru Ca regulation and Biological function the implication of PKC in cell growth was first demonstrated stimulated. J. Biochem..

[80] Okuda H., Adachi M., Miyazawa M., Hinoda Y., Imai K. (1999). Protein kinase Cα promotes apoptotic cell death in gastric cancer cells depending upon loss of anchorage. Oncogene.

[81] Shimizu T., Cao C.X., Shao R.G., Pommier Y. (1998). Lamin B phosphorylation by protein kinase Cα and proteolysis during apoptosis in human leukemia HL60 cells. J. Biol. Chem..

[82] Stransky N., Kim J.L. (2020). PRKC FUSIONS - United States Patent Application Publication.

[83] Dutil E.M., Toker A., Newton A.C. (1998). Regulation of conventional protein kinase C isozymes by phosphoinositide-dependent kinase 1 (PDK-1). Curr. Biol..

[84] Uphoff C.C., Drexler H.G. (2013). Detection of mycoplasma contaminations. Methods Mol. Biol..

[85] Bradford M.M. (1976). A rapid and sensitive method for the quantitation of microgram quantities of protein utilizing the principle of protein-dye binding. Anal. Biochem..

[86] Livak K.J., Schmittgen T.D. (2001). Analysis of relative gene expression data using real-time quantitative PCR and the 2-ΔΔCT method. Methods.

[87] Barber K.W., Miller C.J., Jun J.W., Lou H.J., Turk B.E., Rinehart J. (2018). Kinase substrate Profiling using a Proteome-wide serine-Oriented human Peptide Library. Biochemistry.

