# Supplementary material for: Protein kinase C fusion proteins are paradoxically loss of function in cancer
Source: J Biol Chem. 2021 Feb 20;296:100445. doi: 10.1016/j.jbc.2021.100445 (PMC8008189; doi:10.1016/j.jbc.2021.100445)
Supplement: Figures S1–S5 [file mmc1.pdf]

Supporting Information

Protein kinase C fusion proteins are paradoxically loss of function in cancer

An-Angela N. Van[1,2], Maya T. Kunkel[1], Timothy R. Baffi[1,2], Gema Lordén[1], Corina E. Antal[1,2], Sourav Banerjee[1], and Alexandra C. Newton[1]*

[1]Department of Pharmacology, University of California at San Diego, La Jolla, CA, USA
[2]Biomedical Sciences Graduate Program, University of California at San Diego, La Jolla, CA, USA

*For correspondence: Alexandra C. Newton
E-mail: anewton@health.ucsd.edu

Present address for Corina E. Antal: Gene Expression Laboratory, Salk Institute for Biological Studies, La Jolla, CA 92037, USA
Present address for Sourav Banerjee: Division of Cellular Medicine, School of Medicine, University of Dundee, Dundee DD1 9SY, UK

Supplementary Experimental Procedures

Figure S1: PKC fusion proteins require phosphorylatable residues at priming sites for activity.

Figure S2: Purified GGA2-PKCβII fusion protein is in complex with molecular chaperones and other proteins.

Figure S3: Inhibition of proteasomal or endosomal degradative pathways does not prevent degradation of TANC2-PKCα fusion protein upon cycloheximide treatment.

Figure S4: Knockdown of E3 ligases does not allow for accumulation of the TANC2-PKCα fusion protein in CRISPR-edited cells.

Figure S5: UTP-induced translocation is abolished by disruption of DAG binding.

Table S1: PKC gene fusions identified in cancer. *(.xlsx file)*

Table S2: Recurrent PKC gene fusions in cancer. *(.xlsx file)*

Supplementary Experimental Procedures

Antibodies and reagents

The antibodies used in these supplemental data are as listed: GST (sc-138, Santa Cruz), Hsp70 (610608, BD Transduction), Hsp90 (610418, BD Transduction), Cdc37 (D28H7, 4222S, Cell Signaling), p21 (2947S, Cell Signaling), and CHIP (S1073, Sigma-Aldrich). The RINCK antibody was previously described (57). The reagents used in these data are as listed: MG-132 (474790, Calbiochem), chloroquine (C6628, Sigma-Aldrich), and wortmannin (W1628, Sigma-Aldrich).

Purification of GST-tagged PKC protein from Sf9 insect cells

Human PKCβII and GGA2-PKCβII were subcloned into a pFastBac HT/B vector (Invitrogen) modified with an N-terminal GST tag. Baculovirus was made using the Bac-to-Bac Baculovirus Expression System (Invitrogen). The pFastBac plasmids were transformed into DH10Bac cells, and the resulting bacmid DNA was transfected into Sf9 insect cells (11496015, Thermo Fisher Scientific) in Sf-900 II SFM media (10902-088, Gibco) via CellFECTIN (Thermo Fisher Scientific). The recombinant baculoviruses were harvested and amplified, with Sf9 insect cells grown in shaking cultures at 27 °C. GST-tagged PKCβII and GGA2-PKCβII were batch-purified from baculovirus-infected Sf9 insect cells using glutathione sepharose beads. Sf9 insect cells were infected with baculovirus for either protein and incubated for 3 days in shaking cultures at 27 °C. Infected cells were then pelleted, rinsed in PBS, and lysed in buffer containing 50 mM HEPES (pH 7.5), 1 mM EDTA, 100 mM NaCl, 0.1% Triton X-100, 0.1 mg/mL BSA, 100 μM PMSF, 1 mM DTT, 2 mM benzamidine, 50 μg/mL leupeptin, and 1 μM microcystin. The soluble lysate was then incubated with the glutathione beads for 2.5 h at 4 °C. The beads were washed three times in buffer containing 50 mM HEPES (pH 7.5), 1 mM EDTA, 100 mM NaCl, 0.1 mg/mL BSA, and 1

mM DTT. Protein was eluted in wash buffer supplemented with 10 mM glutathione. The eluted protein was concentrated using 50 kDa Amicon centrifugal filter units (EMD Millipore), exchanged into buffer containing 20 mM HEPES (pH 7.5), 1 mM EDTA, 1 mM EGTA, and 1 mM DTT. An equal volume of glycerol was added, and the protein stock was stored at -20 °C. Protein was quantified utilizing BSA standards run alongside the purified protein on an SDS-PAGE gel stained with Coomassie Brilliant Blue.

Purification of FLAG-tagged PKC protein from HEK 293T cells

HEK 293T cells were transfected with 3XFLAG-tagged PKCβII and GGA2-PKCβII in 15 cm plates via Effectene (301427, QIAGEN) (87). After 48 h incubation, cells were pelleted, rinsed in PBS, and lysed in buffer containing 20 mM Tris (pH 7.5), 150 mM NaCl, 1 mM EDTA, 1 mM EGTA, 1% Triton X-100, 2.5 mM sodium pyrophosphate, 1 mM β-glycerophosphate that was supplemented with 1 mM Na_3VO_4, 1 mM DTT, 1 mM PMSF, 10 μg/mL leupeptin, 1 μM microcystin, and 10 ug/mL aprotinin. Detergent-solubilized lysates were incubated with anti-FLAG M2 affinity gel (A2220, Sigma-Aldrich) for 1 h at 4 °C. Beads were washed twice in lysis buffer and twice in elution buffer, containing 50 mM HEPES (pH 7.4), 0.01% NP40, 100 mM NaCl, 5 mM β-glycerophosphate, 0.1 mM Na_3VO_4, and 10% glycerol. Beads were then incubated in elution buffer containing 0.5 mg/mL of FLAG peptide (F4799, Sigma) for 1 h at 4 °C. The supernatant was divided into aliquots, snap frozen, and stored at -80 °C. Protein was quantified using the Pierce Coomassie (Bradford) Protein Assay Reagent (1856209, Thermo Fisher Scientific): 5 μl of protein or BSA standard was mixed with 45 μl of buffer containing 0.05 M NaOH and 20 mM Tris, 500 μl of the Coomassie reagent was added to the mixture, and the sample

was incubated for 10 min at room temperature. Samples were read via spectrophotometer at 595 nm.

siRNA Knockdown of *TRIM41* (RINCK) and *STUB1* (CHIP)

MDA-MB-231 cells were transfected with 200 nM siRNA for *TRIM41* (RINCK) (L-007105-00-0005, Dharmacon), *STUB1* (CHIP) (L-007201-00-0005, Dharmacon), or both via Lipofectamine 3000 in serum-free media. After 3 h, serum-free media was removed from cells, and complete media was added. Cells were incubated for 72 h and then harvested. Cells were lysed in buffer containing 50 mM Tris (pH 7.4), 1% Triton X-100, 50 mM NaF, 10 mM $Na_4P_2O_7$, 100 mM NaCl, 5 mM EDTA, 1% deoxycholic acid that was supplemented with 1 mM Na_3VO_4, 1 mM phenylmethyl-sulfonyl fluoride, 50 μg/ml leupeptin, 1 μM microcystin, 1 mM DTT, and 2 mM benzamidine. Whole-cell lysates were briefly sonicated and boiled in sample buffer for 5 min at 95 °C.

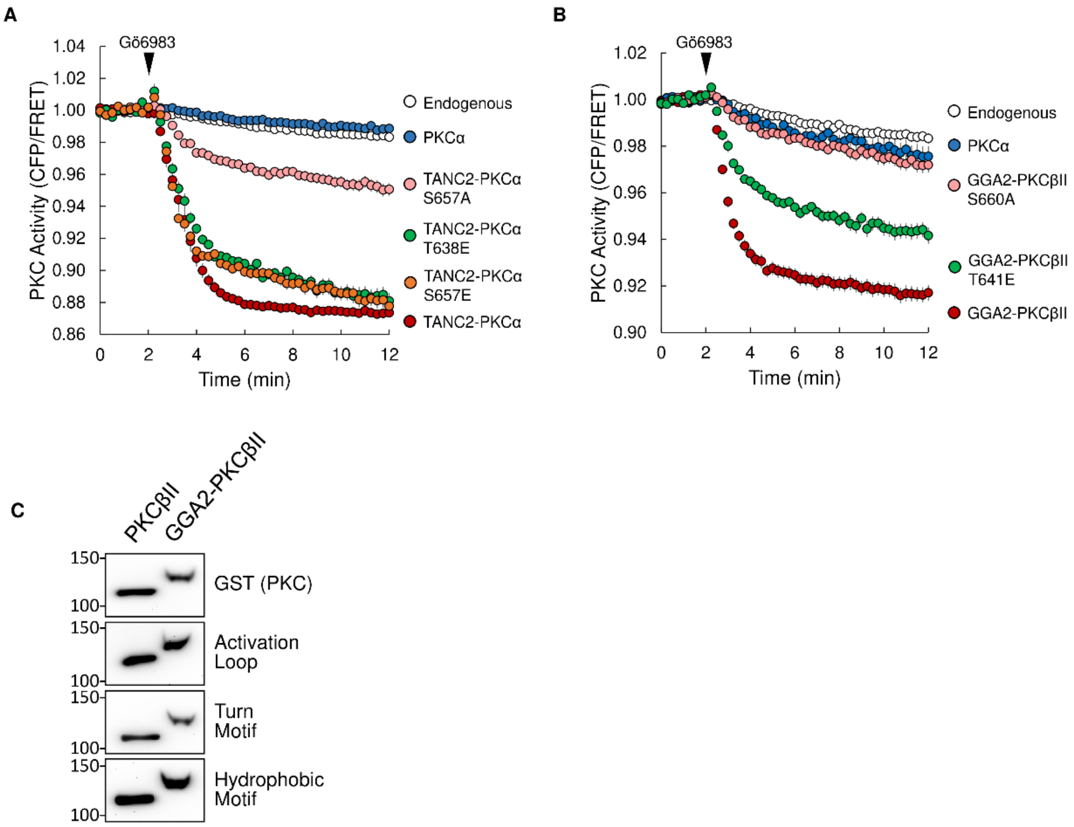

Figure S1: PKC fusion proteins require phosphorylatable residues at priming sites for activity. (**A**) Analysis of basal PKC activity in COS7 cells transfected with CKAR alone (Endogenous) or co-transfected with the indicated mCherry-tagged PKCα construct and treated with inhibitor Gö6983 (1 µM). Constructs of the fusion in which the turn and hydrophobic motif residues are mutated to glutamate (T638E and T657E, respectively) represent constitutive phosphorylation at those sites while mutation to alanine represents an unphosphorylatable site (T657A). Graphs depict normalized FRET ratio changes (mean ± SEM) from three independent experiments. (**B**) Analysis of basal PKC activity in COS7 cells transfected with CKAR alone (Endogenous) or co-transfected with the indicated mCherry-tagged PKCβII construct and treated with inhibitor Gö6983 (1 µM). While the T641E mutant phenocopies constitutive phosphorylation at the turn motif, the S660A mutant cannot be phosphorylated at the hydrophobic motif. Graphs depict normalized FRET ratio changes (mean ± SEM) from three independent experiments. (**C**) Immunoblot analysis of purified wild-type PKCβII or GGA2-PKCβII fusion protein. PKC purified from baculovirus-infected insect cells via GST pulldown and elution with glutathione. Blots probed for total PKC and phosphorylation at priming sites with indicated antibodies.

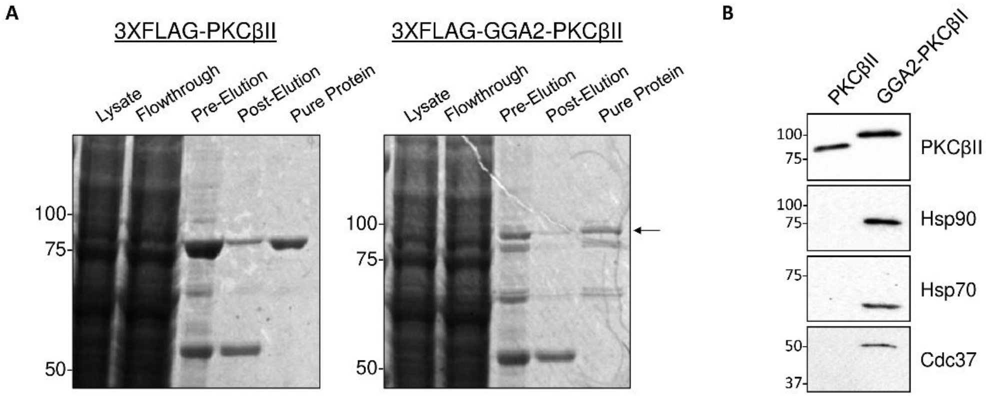

Figure S2: Purified GGA2-PKCβII fusion protein is in complex with molecular chaperones and other proteins. (**A**) Coomassie Blue-stained SDS/PAGE gel of different stages during purification of 3XFLAG-tagged wild-type PKCβII or GGA2-PKCβII fusion protein. Protein purified from HEK 293T cells via FLAG pulldown and elution with FLAG peptide. Arrow indicates band corresponding to fusion protein. (**B**) Immunoblot analysis of purified PKCβII or GGA2-PKCβII protein, probed for PKC or the molecular chaperones Hsp90, Hsp70, and Cdc37.

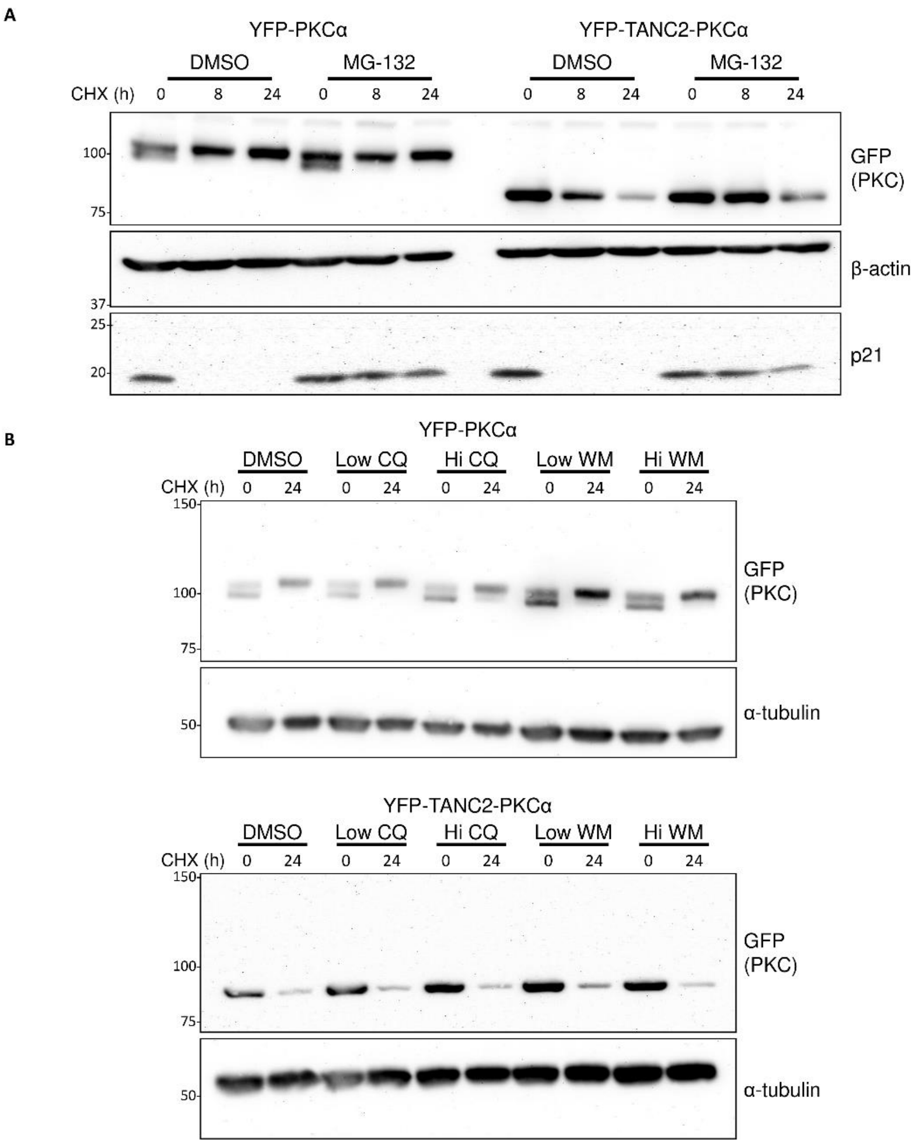

Figure S3: Inhibition of proteasomal or endosomal degradative pathways does not prevent degradation of TANC2-PKCα fusion protein upon cycloheximide treatment. (**A**) Immunoblot analysis of lysates from COS7 cells transfected with YFP-tagged PKCα or TANC2-PKCα, pre-treated with MG-132 (10 μM) or vehicle (DMSO), and treated with cycloheximide (CHX, 355 μM). Cells were harvested at indicated timepoints. Blots probed for GFP, β-actin, and p21. (**B**) Immunoblot analysis of lysates from COS7 cells transfected with YFP-tagged PKCα or TANC2-PKCα, pre-treated with chloroquine (CQ, Low: 100 nM, Hi: 20 μM), wortmannin (WM, Low: 100 nM, Hi: 500 nM), or vehicle (DMSO), and treated with cycloheximide (CHX, 355 μM). Cells were harvested at indicated timepoints. Blots probed for GFP and α-tubulin.

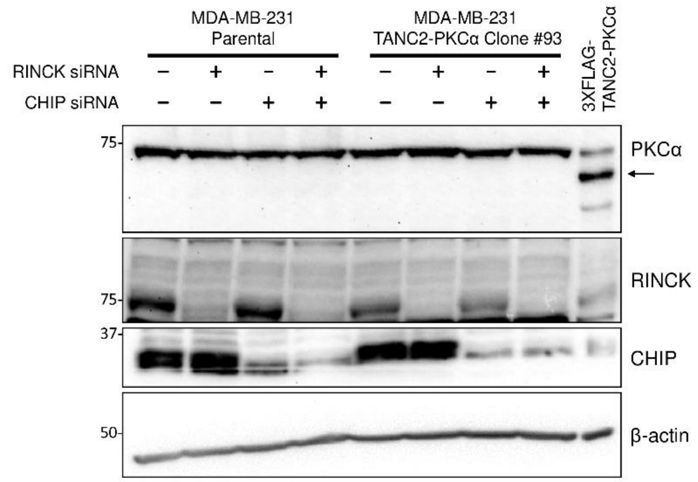

Figure S4: Knockdown of E3 ligases does not allow for accumulation of the TANC2-PKCα fusion protein in CRISPR-edited cells. Immunoblot analysis of lysates from MDA-MB-231 parental and CRISPR-edited TANC2-PKCα fusion-expressing cells. Parental and CRISPR-edited cells were transfected with control siRNA, *TRIM41* (RINCK) siRNA, *STUB1* (CHIP) siRNA, or both *TRIM41* and *STUB1* siRNAs. Lysate from parental MDA-MB-231 cells overexpressing 3XFLAG-tagged fusion protein (arrow) was included as a control. Blots probed for PKCα, RINCK, CHIP, and β-actin. Data is representative from three independent experiments for knockdown of RINCK and CHIP, separately.

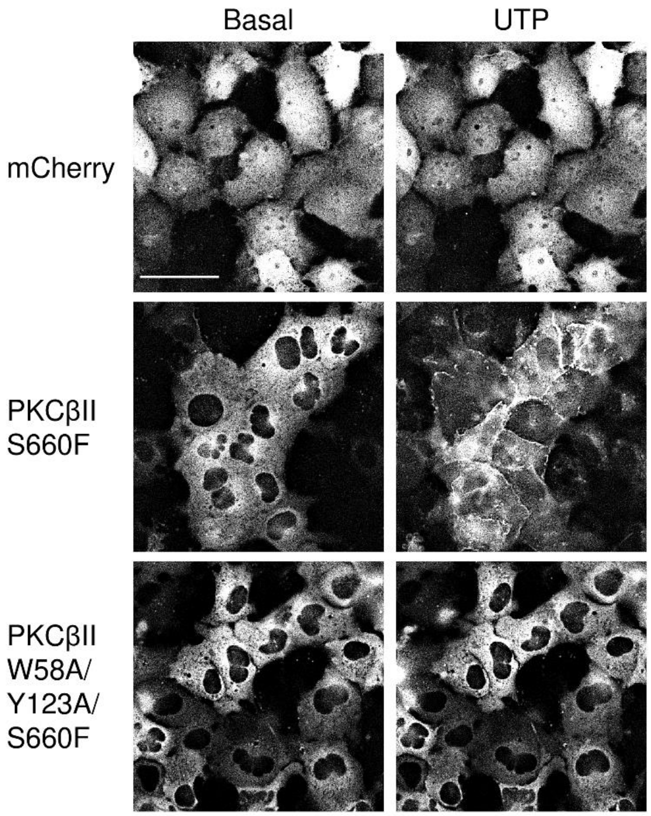

Figure S5: UTP-induced translocation is abolished by disruption of DAG binding. Images of cells from Figure 6C. COS7 cells were transfected with vector or the indicated mCherry-tagged PKC. Images depict mCherry fluorescence following 15 min of UTP stimulation. Scale bar, 50 μm.
